# Targeting oxidative stress-mediated regulated cell death as a vulnerability in cancer

**DOI:** 10.1016/j.redox.2025.103686

**Published:** 2025-05-19

**Authors:** Danyao Chen, Ziyu Guo, Lei Yao, Yuming Sun, Yating Dian, Deze Zhao, Yizhe Ke, Furong Zeng, Chunfang Zhang, Guangtong Deng, Linfeng Li

**Affiliations:** a Department of Dermatology, Xiangya Hospital, Central South University, Changsha, China; b National Engineering Research Center of Personalized Diagnostic and Therapeutic Technology, China; c Furong Laboratory, Changsha, Hunan, China; d Hunan Key Laboratory of Skin Cancer and Psoriasis, Hunan Engineering Research Center of Skin Health and Disease, Xiangya Hospital, Central South University, Changsha, China; e National Clinical Research Center for Geriatric Disorders, Xiangya Hospital, China; f Department of Thoracic Surgery, Xiangya Hospital, Central South University, Changsha, Hunan, China; g Department of Liver Surgery, Xiangya Hospital, Central South University, Changsha, Hunan, China; h Department of Plastic and Cosmetic Surgery, Xiangya Hospital, Central South University, 87 Xiangya Road, Changsha, 410008, Hunan Province, China; i The First Affliated Hospital of Shihezi University, China; j Department of Oncology, Xiangya Hospital, Central South University, Changsha, Hunan, China

## Abstract

Reactive oxygen species (ROS), regulators of cellular behaviors ranging from signaling to cell death, have complex production and control mechanisms to maintain a dynamic redox balance under physiological conditions. Redox imbalance is frequently observed in tumor cells, where ROS within tolerable limits promote oncogenic transformation, while excessive ROS induce a range of regulated cell death (RCD). As such, targeting ROS-mediated regulated cell death as a vulnerability in cancer. However, the precise regulatory networks governing ROS-mediated cancer cell death and their therapeutic applications remain inadequately characterized. In this Review, we first provide a comprehensive overview of the mechanisms underlying ROS production and control within cells, highlighting their dynamic balance. Next, we discuss the paradoxical nature of the redox system in tumor cells, where ROS can promote tumor growth or suppress it, depending on the context. We also systematically explored the role of ROS in tumor signaling pathways and revealed the complex ROS-mediated cross-linking networks in cancer cells. Following this, we focus on the intricate regulation of ROS in RCD and its current applications in cancer therapy. We further summarize the potential of ROS-induced RCD-based therapies, particularly those mediated by drugs targeting specific redox balance mechanisms. Finally, we address the measurement of ROS and oxidative damage in research, discussing existing challenges and future prospects of targeting ROS-mediated RCD in cancer therapy. We hope this review will offer promise for the clinical application of targeting oxidative stress-mediated regulated cell death in cancer therapy.

## 1 Introduction

In aerobic metabolism, cellular redox (oxidation-reduction) reactions predominantly favor oxidation [1]. In response to the pressures of natural selection, organisms have developed precise and efficient reduction systems to counterbalance this tendency [2]. The overall impact of the redox system is determined by the balance between the quantity and type of reactive oxygen species (ROS) and the antioxidant capacity of the organism. Under physiological conditions, redox system stabilize within a dynamic equilibrium known as the "homeodynamic space", where "redox eustress" occurs [3]. In this state, ROS are involved in nearly all physiological activities, contributing to the maintenance of cellular function and survival [4]. However, when redox balance is disrupted, the organism enters a pathological state, which can manifest as subphysiological reductive stress, characterized by an excess of the reduction system that impairs redox signaling, or as supraphysiological oxidative stress, where oxidation exceeds antioxidant defenses, leading to signaling disruption and oxidative damage [1,3].

In tumors, redox imbalance is frequently observed due to high energy demands and metabolic heterogeneity [[5], [6], [7]]. To accommodate these changes, cancer cells exhibit a paradoxical state characterized by the coexistence of elevated ROS production and enhanced antioxidant levels [5,6]. The upregulation of antioxidant defenses increases cancer cells' tolerance to harmful effects and enable dynamic regulation of ROS concentrations to meet the varying demands of different tumor growth stages [8]. Within tolerable limits, oxidative damage can promote oncogenic transformation and contribute to the maintenance of the cancer phenotype [9]. However, when ROS levels exceed a certain threshold, excessive oxidative damage becomes lethal to cancer cells, inducing various forms of cell death. Based on whether cell death is regulated by specific molecular mechanisms, it can be broadly classified into instantaneous and catastrophic accidental cell death caused by sudden, uncontrollable external physical or chemical factors, and regulated cell death (RCD), which is governed by intricate molecular signals and mechanisms within the cell [10]. Increasing studies have demonstrated that ROS are involved in the complex regulation of various forms of RCD, highlighting that ROS-mediated RCD holds potential applications in cancer therapy.

Recently, researchers have been slowly realizing that the interplay between intrinsic gene expression, developmental stage, mutational status of tumor cells, and varying levels of ROS and their harmful effects ultimately determines the type of RCD that occurs [11]. However, there remains a lack of comprehensive summaries elucidating how ROS modulate various forms of RCD and their applications in cancer treatment. In this review, we first provide a comprehensive overview of the mechanisms underlying ROS production and control within cells, highlighting their dynamic balance. Next, we discuss the paradoxical nature of the redox system in tumor cells, where ROS can promote tumor growth or suppress it, depending on the context. We also systematically explored the role of ROS in tumor signaling pathways and revealed the complex ROS-mediated cross-linking networks in cancer cells. Following this, we focus on the intricate regulation of ROS in RCD and its current applications in cancer therapy. We further summarize the potential of ROS-induced RCD-based therapies, particularly those mediated by drugs targeting specific redox balance mechanisms. Finally, we address the measurement of ROS and oxidative damage in research, discussing existing challenges and future prospects of targeting ROS-mediated RCD in cancer therapy.

### 1.1 Source and control of cellular ROS

**Fig. 1:**
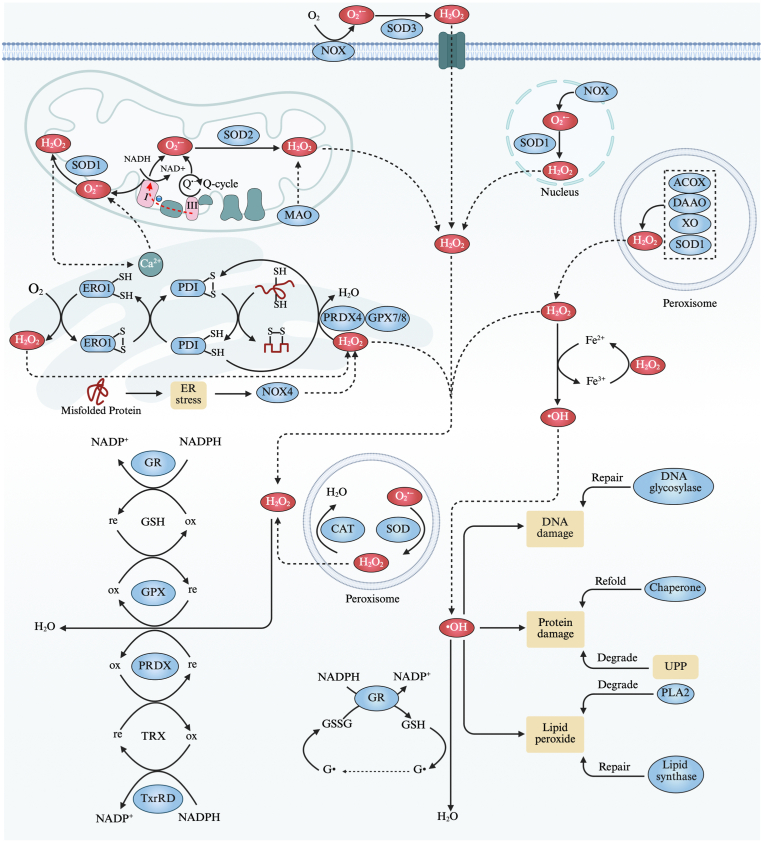
Source and control of cellular ROS. The upper section depicts major ROS sources, including mitochondria, NOXs, ER and Fenton reaction. In mitochondria, ROS is mainly derived from the reverse electron transfer at complex I and semiquinone-dependent Q-cycle at complex III, with a smaller fraction produced as byproducts of ETC. In the ER, ROS is generated during protein folding catalyzed by PDI and ERO1 with the release of H_2_O_2_ and ER stress which activates NOX4 and promotes calcium release to stimulate the overproduction of mitochondrial ROS. Fenton reaction produces •OH through the reaction of Fe^2+^ with H_2_O_2_, exacerbating biomolecular damage. And additional ROS sources include enzymes like ACOX, DAAO, XO and MAO. The lower section focuses on ROS detoxification and repair systems. Antioxidant enzymes, including SODs, CAT, GPX, PRDX, work in concert with the GSH and thioredoxin TRX systems to neutralize ROS. Excessive ROS, which lead to oxidative damage of DNA, proteins, and lipids, is mitigated through dedicated repair pathways.

ROS is a general term for a group of molecules derived from oxygen that have vastly different reactions, including superoxide anion radical (O_2_^•−^), hydroxyl radical (•OH), peroxyl radical (ROO•), alkoxyl radical (RO•), hydrogen peroxide (H_2_O_2_), hypohalous acids (HOX), organic hydroperoxides (ROOH) [12] **(**BOX 1**)**. In redox homeodynamics [1], ROS are rapidly and continuously generating, diffusing and engaging in biochemical reactions. This process is accompanied by the antioxidant system that specifically regulates and maintains redox signaling within physiological limits, thereby preventing oxidative damage [13,14]. To provide a deeper and more comprehensive understanding, we will discuss the highly regulated dynamic process of cellular ROS from its sources to its destinations (Fig. 1).

BOX 1 **Terminology of reactive oxygen species and their characteristics**

**Superoxide anion radical (O_2_^•−^)**

O_2_^•−^ is produced by single-electron reduction of oxygen, mainly from electron leakage in the process of electron transport chain (ETC) and the reaction of NADPH and O_2_ catalyzed by NADPH oxidases. A major fate is dismutation to produce H_2_O_2_ and O_2_, serving as the main source of H_2_O_2_. Under the high electrostatic attraction, O_2_^•−^ rapidly reacts with Fe–S clusters, releasing iron. The protonated form,HO_2_, can diffuse within lipids and has the ability to extract hydrogen atoms from polyunsaturated fatty acids (PUFAs), creating carbon-centered radicals.

**Hydroxyl radical (•OH)**

•OH is an exceptionally toxic oxidant that reacts indiscriminately with biological molecules. It is primarily generated through Fenton chemistry, which involves the Fenton reaction mediated by Fe^2+^ and H_2_O_2_, simultaneously influencing the toxicity range of •OH through the position of Fe^2+^. Due to the high reactivity and instantaneous nature of •OH, it is difficult to eliminate and has almost no direct signaling function [4].

**Peroxyl radical (ROO•)**

ROO•, primarily originating from the free radical chain reactions of PUFAs. As a crucial intermediate, it propagates the lipid peroxidation reaction chain by abstracting hydrogen atoms from other lipid molecules.

**Alkoxyl radical (RO•)**

RO•, another intermediate of lipid peroxidation, is further catalyzed from organic hydroperoxides in the presence of transition metal catalysts. Compared to ROO•, RO• exhibits a higher oxidation activity, further amplifying the chain reaction.

**Hydrogen peroxide (H_2_O_2_)**

H_2_O_2_, mainly produced by O_2_^•−^ dismutation reaction and NADPH oxidases, is relatively stable and can traverse membranes through aquaporins, providing greater ROS signal transmission capability [88]. While H_2_O_2_ has weak oxidative activity, it can rapidly react with Fe^2+^ to generate the extraordinarily strong oxidant •OH, leading to more severe oxidative damage. The decomposition of H_2_O_2_ is primarily mediated by a series of enzymes, including catalase, glutathione peroxidases, and peroxiredoxins, which help maintain appropriate H_2_O_2_ concentrations and stabilize its signaling activity.

**Hypohalous acids (HOX)**

HOX is primarily generated by phagocytic cell myeloperases catalyzing the reaction between H_2_O_2_ and halogen ions (X^−^, mainly including Cl^−^ and Br^−^), and participates in killing invading pathogens in phagolysosome. HOX can also be released extracellularly to participate in intercellular signaling.

**Organic hydroperoxides (ROOH)**

ROOH, an intermediate product of lipid peroxidation, is also generated through lipid oxidation catalyzed by enzymes such as lipoxygenases and cyclooxygenases. It plays a significant role in cell signal transduction and is involved in the regulation of cell proliferation, apoptosis and inflammation responses.

Alt-text: BOX 1

### 1.2 Endogenous and exogenous ROS source

***Leaking of electrons in mitochondrial electron transport chain (ETC).*** In the late 1950s and early 1960s, research on mitochondrial ROS production began to emerge [15,16]. Later, in 1971–1973, Chance et al. elucidated that the mitochondrial ETC should be regarded as a significant intracellular source of ROS [17,18], sparking a surge of research in this field [[19], [20], [21]]. Currently, the mitochondrial ETC is widely identified as a primary source of ROS, with reverse electron transfer at complex I and semiquinone-dependent reaction at complex III being the main contributors [20,22,23]. Specifically, under normal physiological conditions, mitochondria efficiently generate energy through complex V, which captures and converts electrochemical potential of proton gradient established by the forward electron transfer. This process involves a cascade of electron transfers initiated by complex I with electrons derived from nicotinamide adenine dinucleotide hydrogen (NADH) [12,24]. Throughout this process, although the generation of ROS is inevitable, the amount produced remains minimal [[25], [26], [27], [28]]. However, when ubiquinone accumulates excessively or complex V experiences activation disorders, reverse electron transport occurs, where electrons flow back through complex I, reducing NAD^+^ into NADH and producing highest proportion of ROS [29,30]. Thus, complex I is recognized as the largest contributor to ROS production, although highly controversial [24,[31], [32], [33]], mainly because highly variable and difficult-to-measure factors influencing ROS production in vivo make it unachievable to evaluate ROS levels through isolated mitochondria [25]. Complex III also plays a critical role, being recognized as the second-largest contributor to ROS production within the mitochondrial system [34,35]. The electron transfer process at complex III is facilitated by the Q-cycle, during which complex III acquires electrons from fully reduced ubiquinone, subsequently reducing cytochrome *c* to help maintain the transmembrane proton gradient [36,37]. Concurrently, in the outer ubiquinone-binding site of complex III, half of the electrons are diverted along an alternative pathway to generate highly reactive ubisemiquinone, which can migrate freely within complex III and directly leak a single electron to oxygen, leading to ROS production [[38], [39], [40], [41]]. Interestingly, the ROS produced by mitochondria also play a regulatory role in mitochondrial morphology and function through redox signaling, highlighting the intricate interplay essential for cellular health and functionality [42].

***ROS-generating NADPH oxidases (NOXs).*** NOXs are considered to be another major source of ROS. Unlike other sources, NOXs are an enzymatic system whose primary function is the specific production of ROS rather than serving as a mere byproduct [43]. The NOX family comprises seven isoforms (NOX1-NOX5 and DUOX1/2), all of which are transmembrane proteins that deliver electrons from NADPH to extracellular oxygen, resulting in the generation of superoxide or H_2_O_2_ in various cells and tissues [44,45]. ROS produced by NOXs can further activate other oxidase system, leading to sustained oxidative stress and tissue injury [46,47]. Typically, NOXs are activated in a regulated manner in response to stimuli such as growth factors, cytokines, and calcium [48,49]. The electron transfer occurs through two key catalytic domains: the dehydrogenase domain and the transmembrane domain, where electrons are sequentially transferred from NADPH to oxygen via linear flavin adenine dinucleotide and inner and outer heme groups [50,51]. Subsequently, the H_2_O_2_ produced can traverse biological membranes, particularly through aquaporins, enabling it to transmit redox signals over considerable distances [52,53]. Recent studies also proposed a new hypothesis suggesting that the activation of NOXs requires relative movement of the NADPH-binding domain toward the flavin adenine dinucleotide-binding domain to form a productive topology within the dehydrogenase domain after the assembly of stimuli [44]. This hypothesis was further validated by the research of Liu et al. [54]. However, the precise mechanism by which stimuli activate NOXs remains incompletely elucidated. Advancements in structural information about NOXs will provide a foundation for more comprehensive investigations into their activation mechanisms [54].

***Byproducts of protein folding within the endoplasmic reticulum (ER) and ER stress.*** As a central hub for protein processing, the ER regulates the precise folding and post-translational modifications of newly synthesized proteins [55,56]. This process typically involves the extensive formation of disulfide bonds, primarily catalyzed by protein disulfide isomerase (PDI). Concurrently, large amounts of H_2_O_2_ are produced due to the re-oxidation of PDI by endoplasmic reticulum oxidoreductase 1 (ERO1), through a series of thiol-disulfide exchange reactions that culminate in electron transfer to oxygen [57]. Subsequently, H_2_O_2_ serves as a substrate for peroxiredoxin 4 (PRDX4), glutathione peroxidase 7 (GPX7), and glutathione peroxidase 8 (GPX8), further promoting the enzymatic oxidative protein folding process [58]. However, not all proteins are correctly folded and efficiently transported to their designated organelles within the requisite timeframe. When ER homeostasis and proper protein folding are disrupted, the accumulation of unfolded and misfolded proteins can exceed the capacity of ER-associated degradation (ERAD), leading to ER stress [59]. In response, the unfolded protein response (UPR) is initiated—a series of cellular mechanisms aimed at restoring ER homeostasis by reducing protein synthesis, upregulating molecular chaperones to enhance protein folding, and improving protein degradation via ERAD [56,60]. This response directly leads to the activation and increased expression of NOX4, further contributing to the cellular ROS pool [61]. Additionally, ER stress can disrupt calcium homeostasis, resulting in the release of calcium from the ER into the cytoplasm. Elevated cytosolic calcium levels can enhance the activity of mitochondrial enzymes with mitochondrial calcium intake increases, leading to an overproduction of ROS [62]. Increased mitochondrial ROS signaling can feed back into the ER, further triggering calcium release and ultimately resulting in a vicious cycle [62].

***Other less significant endogenous sources***. In addition to the major sources of ROS previously discussed, there are several less prominent yet noteworthy endogenous sources of ROS. For example, cytochrome P450 enzymes generate ROS as byproducts during the metabolism of various substrates, including drugs and toxins [63]. Peroxisomes can also produce H_2_O_2_ as a metabolic byproduct from the fatty acid beta-oxidation mediated by acyl-coenzyme A oxidase (ACOX) and the oxidation of d-amino acids mediated by d-amino acid oxidase (DAAO) [64,65]. Additionally, enzymes such as monoamine oxidase (MAO) and xanthine oxidase (XO) directly produce H_2_O_2_ [66,67]. Cyclooxygenase contributes to ROS generation as intermediates to initiate and regulate inflammatory responses [68]. Although these sources may represent relatively minor contributions to overall ROS levels, they play crucial roles in specific cellular contexts and can significantly impact homeostasis and the occurrence of diseases.

***Some highly variable exogenous ROS sources.*** Exogenous ROS sources primarily stem from cumulative environmental exposures, including ultraviolet light, ionizing radiation and contaminant [69]. Upon ultraviolet light irradiation, photosensitizers or chromophores in the skin (e.g., cytochromes, heme, and porphyrins) absorb photons and become excited [70]. These excited molecules then react with oxygen to generate ROS, primarily including O_2_^•−^ and singlet oxygen (^1^O_2_) [70]. Ionizing radiation can penetrate living cells and primarily generates ROS through the radiolysis of intracellular water, given the high aqueous content of biological tissues [71]. Contaminants, including air pollutants (such as particulate matter, nitrogen dioxide, and ozone), chemicals (such as heavy metals, pesticides, herbicides, and industrial compounds like polycyclic aromatic hydrocarbons), can trigger the activation of immune cells to produce ROS as part of the defense response [72,73]. Additionally, these contaminants may interfere with the Eleading to electron leakage and subsequent ROS generation [74,75]. Given the extensive variability in environmental exposures, the generation of exogenous ROS is highly inconsistent and challenging to quantify and regulate.

### 1.3 Amplification of ROS

***Fenton chemistry.*** Hydroxyl radicals (•OH) are exceptionally potent and highly toxic oxidants characterized by their indiscriminate reactivity towards biological molecules. They are primarily generated through Fenton chemistry, which includes both the Fenton reaction and Fenton-like reactions [76]. Under appropriate pH conditions (approximately pH 3–4), the reaction is activated when Fe^2+^ encounters sufficient amounts of H_2_O_2_, resulting in the production of Fe^3+^ and •OH. Subsequently, Fe^3+^ can be reduced back to Fe^2+^, allowing the cycle to continue. This constitutes the core mechanism of the Fenton reaction [77]. The Fenton-like reaction expands upon this concept by utilizing not only Fe^2+^ and H_2_O_2_ but also other transition metals (such as Cu^2+^, Co^2+^, Mn^2+^, Ni^2+^, etc.) and alternative oxidants (such as persulfate and peroxymonosulfate) to generate •OH [77]. In summary, Fenton chemistry can significantly amplify the toxicity of ROS. However, it is a double-edged sword: while it provides protection by targeting and eliminating pathogens and harmful cells, it can also induce oxidative stress and damage to normal cellular structures. On the positive side, exploration of Fenton reaction-based chemodynamic therapy is gaining momentum as a promising approach for cancer treatment, aiming to deliver effective therapy with minimal side effects [78,79].

***lipid peroxidation.*** Lipid-derived ROS arise from lipid peroxidation, a chain reaction initiated when ROS attack polyunsaturated fatty acids (PUFAs), leading to the formation of lipid radicals and hydroperoxides [12]. Specifically, •OH extract hydrogen atoms from PUFAs, resulting in the formation of lipid radicals (L•), which then rapidly react with oxygen to produce lipid peroxyl radicals (LOO•) [80]. This propagates a chain reaction, further producing lipid hydroperoxide (LOOH) [80]. And the cycle is perpetuated when LOOH is catalyzed by transition metal ions to form reactive lipid intermediates [80].

### 1.4 Control of cellular ROS and its damage

To address the excessive production and accumulation of the aforementioned ROS to a physiological state, a sophisticated redox control system is activated accordingly and persists throughout the entire process. Focusing on the production process of ROS, we can simplistically consider that superoxide anion (O_2_^•−^) and H_2_O_2_ are initially produced, followed by further reactions that generate peroxynitrite (ONOO^−^), hypohalous acids (HOX), and •OH, which constitutes the second stage [14]. Consequently, based on the different defense mechanisms that operate before, during, and after oxidative injury occurs, the dynamic antioxidant process can be categorized into three phases: the prevention phase, the interception phase, and the repair phase.

***Prevention phase.*** In the prevention phase, the primary antioxidant activities involve the scavenging of O_2_^•−^and H_2_O_2_ through the dismutation of O_2_^•−^ by superoxide dismutases (SODs) and the decomposition of H_2_O_2_ by catalase (CAT), glutathione peroxidases (GPXs), and peroxiredoxins (PRDXs), forming the first line of defense against oxidative stress [[81], [82], [83], [84]]. Under normal circumstances, O_2_^•−^ undergoes spontaneous dismutation mildly to produce H_2_O_2_ and oxygen, a process that is significantly accelerated by SODs [82]. As the only enzymes specifically targeting superoxide, SODs possess strict subcellular localization, balancing ROS in different compartments and controlling overall redox signaling. They can be categorized into three types: copper/zinc SOD1 in the cytoplasm, manganese SOD2 in the mitochondrial matrix, and extracellular copper/zinc SOD3 [[85], [86], [87]]. Notably, this dismutation reaction plays an indispensable role in maintaining optimal H_2_O_2_ concentrations and subsequent stability of ROS signaling [88]. Currently, SOD mimetics are under extensive study and are guiding clinical treatments [85,89]. The elimination of H_2_O_2_ is highly controlled by various enzymatic systems, with water being the only byproduct of its reduction. CAT, an enzyme commonly found in all organisms, primarily resides in peroxisomes, with partial presence in mitochondria and the cytoplasm [90]. Under normal circumstances, H_2_O_2_ is generally decomposed by GPXs (which rely on glutathione (GSH) and GSH reductase (GR) in the presence of NADPH reducing system) and PRDXs (which rely on thioredoxin (Trx) and Trx reductase (TxrRD) in the presence of NADPH reducing system) due to their higher affinity for H_2_O_2_ compared to CAT, while CAT plays a greater contribution in conditions of high H_2_O_2_ concentration [91]. Notably, considering H_2_O_2_'s significant role in signaling pathways, which may outweigh its mild toxicity to organisms, controlling H_2_O_2_ concentrations may be aimed at modulating its signaling activity rather than mitigating its potential toxicity [88].

***Interception phase.*** In the interception phase, the primary objective of antioxidants is to rapidly neutralize already formed free radicals, particularly •OH, to prevent cellular damage. The glutathione and thioredoxin systems are the main contributors to this defense. Central to the glutathione system is glutathione (GSH), the most abundant and significant tripeptide thiol antioxidant synthesized within cells, which also serves as an indicator of oxidative stress based on its intracellular concentration [92,93]. GSH is synthesized sequentially via two enzyme-catalyzed reactions by γ-glutamylcysteine synthetase and glutathione synthetase, and exists either in a reduced thiol form (GSH) or an oxidized disulfide form (GSSG) [94]. Under normal conditions, the majority of glutathione remains in its reduced form, due to the high activity of glutathione reductase (GR) [95,96]. Reduced GSH neutralizes •OH by donating hydrogen and electrons, reducing the radical to water while itself being oxidized to a glutathione radical, which subsequently dimerizes to form GSSG. With the assistance of the coenzyme NADPH, GSSG is reduced back to GSH through the action of GR, enabling the continued antioxidant function of GSH [95]. The thioredoxin system, composed mainly of thioredoxin (Trx) and thioredoxin reductase, plays a complementary or alternative role to the glutathione system [95,97,98]. However, in most cases, both systems are thought to operate concurrently, and the survival of mammals depends on the presence of both [97].

***Repair Phase.*** Unfortunately, once the accumulation of oxidants reaches a certain threshold, inevitable damage occurs, primarily targeting DNA, proteins and lipids [99]. The repair phase involves restoring damaged molecules or eliminating irreparable damage. In most instances, oxidative DNA damage is addressed by several overlapping repair mechanisms that provide multiple layers of protection, mainly including base excision repair (which corrects small, non-helical distorting base damage initiated by DNA glycosylases), nucleotide excision repair (which handles large, helical distortions), homologous recombination and non-homologous end joining (which repairs DNA double-strand breaks) [100,101]. Regarding oxidative damage to proteins, a triage mechanism identifies and evaluates damaged proteins, and then based on the relative activities of ubiquitination and deubiquitination, denatured proteins are either refolded by chaperones or selectively degraded via the ubiquitin-proteasome pathway [102]. When oxidation attacks lipid molecules, particularly unsaturated fatty acids in cell membranes, it triggers a lipid peroxidation cascade, leading to more powerful destruction [80,93]. The body primarily degrades damaged lipids via phospholipase A2 (PLA2) and repairs the cell membrane by re-synthesizing lipids through lipid synthases [103]. Ultimately, under selective pressures, oxidative and antioxidative processes reach a dynamic equilibrium through intrinsic feedback mechanisms, especially under sustained oxidative stress [102,104].

## 2 ROS and cancer

**Fig. 2:**
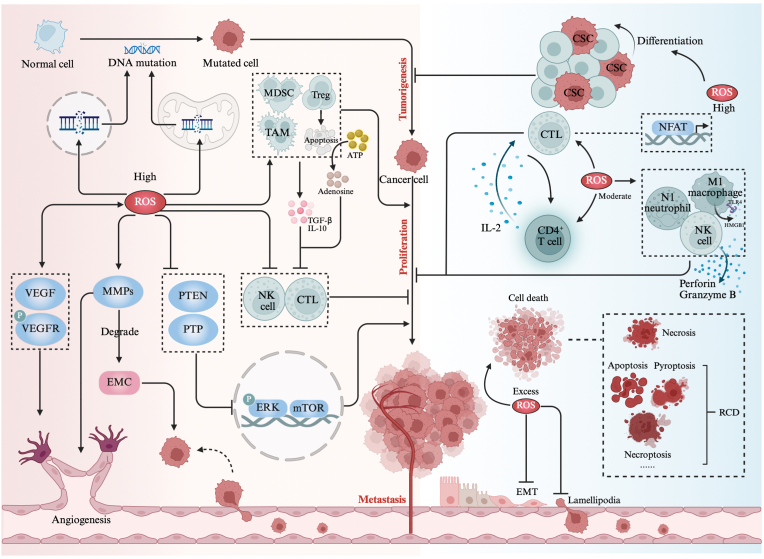
Mechanisms of ROS in tumor promotion and inhibition. High ROS levels promote tumorigenesis through driving DNA mutations to transform normal cells into mutated cells, promote proliferation through inactivating PTEN and PTPs to enhance the ERK and mTOR signaling, and activate VEGF and MMPs to promote angiogenesis and metastasis. High ROS levels also promote an immunosuppressive environment by supporting immunosuppressive cells such as TAMs, Tregs, and MDSCs, collectively inhibiting NK cells and CTLs. Conversely, moderate ROS activate T cells, N1 type neutrophils, NK cells and M1type macrophages, enhancing antitumor immunity. High ROS levels promote CSCs differentiation, exerting tumorigenesis suppression. Excessive ROS accumulation in cancer cells triggers various forms of cell death.

Unlike normal cells, cancer cells exhibit a paradoxical phenotype characterized by concurrently elevated ROS production and upregulated antioxidant defense mechanisms. This phenomenon is attributed to the high energy demands and the heterogeneity of metabolites (including oxygenation, pH, and glucose) resulting from aberrant proliferation and abnormal vasculature, along with the metabolic reprogramming required to provide sufficient ATP and alterations in signaling pathways related to cellular metabolism [5,6]. Notably, ROS play a dual role in tumor promotion and inhibition, depending on its concentration and the stage of cancer progression [105,106] (Fig. 2).

### 2.1 Tumor-promoting functions of ROS

Tumorigenesis is widely recognized to originate from gene mutations that activate oncogenes or inactivate tumor suppressor genes, primarily induced by DNA damage, which can lead to the accumulation of mutagenic 8-oxo-7-hydro-2′- deoxyguanosine (that is poorly repaired and can mediate spontaneous mutation) [107]. This process is exacerbated by cytoplasmic ROS which penetrate the nucleus, causing genomic instability and mutations during DNA replication [104]. Additionally, the mitochondrial genome, due to its vulnerability of location to damage and limited proofreading capabilities, is particularly susceptible to ROS, exhibiting a significantly higher mutation frequency compared to nuclear DNA [9]. Many tumors demonstrate that mitochondrial DNA mutations act as co-initiators alongside nuclear DNA mutations in tumorigenesis [[108], [109], [110]]. Certain mitochondrial DNA mutations are even specific to tumors [111].

During the tumor proliferation phase, ROS can target key proteins, especially the oxidative inactivation of phosphatases such as phosphatase and tensin homolog (PTEN) and protein tyrosine phosphatases (PTPs) [112]. This inactivation enhances the MAPK/ERK and PI3K/Akt/mTOR signaling pathways by maintaining sustained phosphorylation of key proteins, promoting cancer cell proliferation and survival [113,114]. Additionally, within the TME, immune surveillance and clearance, mainly mediated by natural killer (NK) cells and cytotoxic T lymphocytes (CTLs), are also critical factors in tumor growth control [115]. Notably, high ROS levels often induce an immunosuppressive environment that promotes cancer proliferation by enhancing the proliferation, differentiation, and recruitment of immunosuppressive cells to the tumor microenvironment, including myeloid-derived suppressor cells (MDSCs), regulatory T cells (Tregs), and tumor-associated macrophages (TAMs), and collectively leading to the suppression of NK cells and CTLs through direct ROS-induced damage and the release of immunosuppressive cytokines such as IL-10 and TGF-β [116,117]. Notably, Tregs are particularly susceptible to ROS-induced apoptosis due to their deficient NRF2 antioxidant system [107], while apoptotic Tregs exhibit stronger immunosuppressive activity than their live counterparts by converting more ATP to adenosine through the activation of highly expressed CD39 and CD73, even counteract antitumor T cell immunity mediated by programmed death-ligand 1 blockade therapy [118].

Regarding tumor metastasis, ROS play a significant role in neovascularization, particularly in hypoxic environments that stabilize hypoxia-inducible factors (HIFs). Specifically, H_2_O_2_ derived from NOXs increases the expression of vascular endothelial growth factor (VEGF) and induces autophosphorylation of the VEGF receptor, a critical switch for angiogenesis [119]. Simultaneously, HIFs upregulate the transcription of these angiogenic factors [104,120]. VEGF also stimulates NOXs to produce more ROS, creating a feedback loop that amplifies downstream redox signaling, thereby further promotes angiogenesis [119]. Additionally, ROS activate matrix metalloproteinases (MMPs), which are prerequisites for vascular growth [121]. Among them, MMP-9 contributes to tumor cell migration and metastasis by degrading the extracellular matrix (ECM), regulating cell-cell and cell-ECM interactions, and releasing pro-invasive factors [122,123].

### 2.2 Tumor-suppressing functions of ROS

As mentioned above, high accumulation of ROS promotes tumor progression. However, during various stages of tumor development, when ROS levels exceed the adaptive capacity of tumor cells, it induces severe cellular damage, triggering various forms of cell death, including accidental cell death and RCD. Accidental cell death is an instantaneous and catastrophic form of cellular demise, triggered by abrupt and uncontrollable external physical or chemical stimuli, primarily encompassing necrosis. RCD encompasses apoptosis [124], ferroptosis [125], pyroptosis [126], cuproptosis [127], necroptosis [128], parthanatos [129], paraptosis [130], lysosome-dependent cell death [131], and oxeiptosis [132], all intricately modulated by ROS. Due to the characteristics that can be regulated by intricate molecular signals and mechanisms within the cell [10], RCD holds potential applications in cancer treatment.

Regarding tumorigenesis, cancer stem cells (CSCs), which are responsible for initiating tumors and contributing to intratumor heterogeneity, require low ROS levels to maintain their stemness, a condition typically associated with early tumor growth [99]. High ROS levels can promote the differentiation of CSCs by downregulating Hedgehog pathway, thereby causing tumor suppression [133]. During the tumor proliferation phase, within the TME, appropriate ROS concentration is crucial for the activation of T cells (CD4^+^ T cells and CTL), which are key effectors of anti-tumor immunity [115]. Specifically, moderate ROS, particularly those derived from mitochondria, can induce the activation of transcription factor nuclear factor of activated T-cells (NFAT), facilitating T cell activation and subsequently promoting IL-2 secretion which further supports T cell proliferation and function [134]. ROS also enhance the cytolytic activity of NK cells by promoting the release of perforin and granzyme B [135,136], and promote the recruitment and activation of anti-tumor N1 type neutrophils, further exerting anti-tumor effect [137,138]. Besides, Vadevoo et al. suggested that ROS could mediate reprogramming of M2 type macrophages into M1 type macrophages through HMGB1-TLR4 axis stimulation [139], which is well-established by its anti-tumoral effect. For a more comprehensive and detailed understanding of the effects of ROS on tumor proliferation through their impact on the immune system, we recommend consulting several recent reviews in the field [138,[140], [141], [142]]. During tumor metastasis, excess ROS can disrupt the integrity of lipid rafts, thereby impairing the formation of lamellipodia, which are essential for tumor cell migration [143,144]. Additionally, in breast cancer, excess ROS can inhibit epithelial-mesenchymal transition (EMT) by regulating the miR-200c/ZEB1 axis, thereby suppressing tumor metastasis [145].

## 3 Redox signaling in cancer

The impact of ROS on tumors is primarily mediated through a series of redox signaling pathways, which mainly include NRF2 signaling, p53 signaling, hypoxia signaling, FOXO signaling, NF-κB signaling and MAPK/ERK pathway. We will specifically describe the association between ROS and these pathways.

**Fig. 3:**
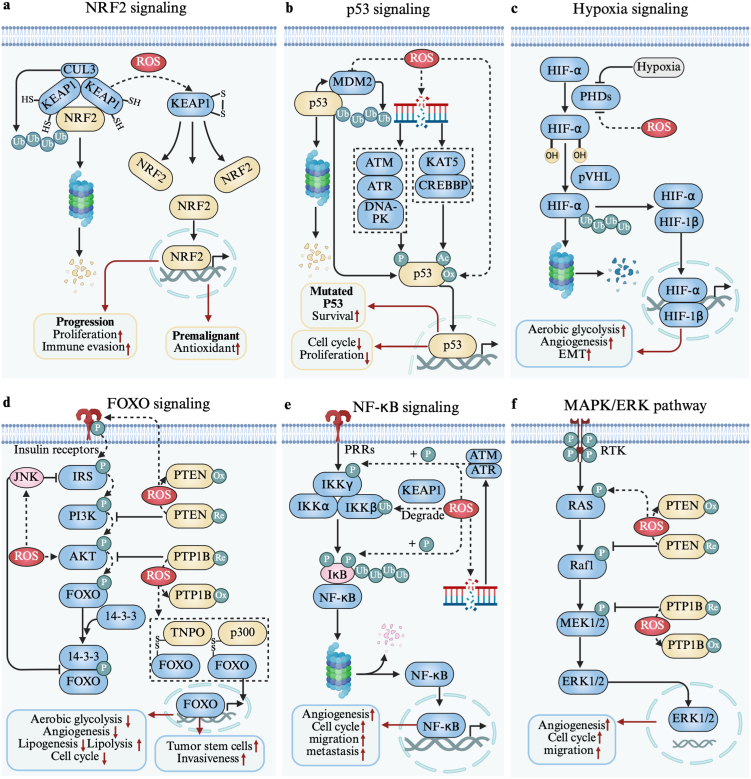
Redox signaling in cancer. **a** ROS activate NRF2 by disrupting KEAP1, leading to transcriptional upregulation of antioxidant genes in premalignant stage to inhibit tumor and proliferation-related genes in progression stage to promotes tumor. **b** ROS inhibit MDM2 activity and its P53 binding site or directly modify p53 to stabilize p53, driving cell cycle arrest and proliferation suppression. **c** ROS inhibit PHDs stabilize HIF-α, promoting glycolysis, angiogenesis, and EMT. **d** ROS activate JNK signaling, which reduces IRS activity and dissociates FOXO from 14-3-3, oxidize and inactivate the phosphatases PTP1B and PTEN, promote the formation of heterodimers with transportin (TNPO) and acetyltransferase p300, thereby activating FOXO and regulating cellular metabolism, stem cell maintenance, and invasiveness. **e** NF-κB signaling is subject to dual regulation by ROS. ROS phosphorylate IKKγ and IκB to activate NF-κB signaling. ROS mediate the degradation of IKKβ, inhibiting NF-κB signaling. **f** ROS enhance MAPK/ERK pathway activity through inactivation of PTEN and PTP1B, supporting cell proliferation, migration, and angiogenesis.

***NRF2 signaling.*** NRF2 is a master transcription factor involved in regulating antioxidant and detoxification responses [146]. Under normal physiological conditions, NRF2 is tightly controlled by Keap1 in the cytoplasm. Mechanistically, two homodimerized KEAP1 molecules bridge an E3 ubiquitin ligase complex by binding to CULLIN3 (CUL3). Acting as a substrate adapter, KEAP1 interacts with one NRF2 through two binding motifs in the NRF2-ECH homology 2 domain, namely DLG and ETGE. This interaction mediates the conjugation of seven lysine residues between these motifs with ubiquitin. Subsequently, polyubiquitin-labeled NRF2 is transported to the 26S proteasome for rapid degradation [147]. However, a small fraction of NRF2 escapes KEAP1-CUL3-mediated ubiquitination and degradation, maintaining basal antioxidant activity while preventing unnecessary activation of antioxidant genes [148]. Upon oxidative stress, modifications of critical cysteine residues in Keap1 (formation of intramolecular disulfide) induce a conformational change, disrupting the ubiquitin-proteasome degradation pathway [149]. This allows NRF2 to accumulate in the cytoplasm, after which it translocates to the nucleus to bind antioxidant response elements on DNA and activate the transcription of over 200 target genes [150] (Fig. 3a).

In cancer, NRF2 exhibits a dual regulatory role: it acts as a tumor suppressor in the premalignant stages but promotes tumorigenesis and mediates treatment resistance once cancer has developed [151,152]. Due to the unique oxidative environment in tumors, NRF2 is often constitutively activated, with somatic mutations at the NRF2-Keap1 binding sites frequently observed [148,153]. NRF2 induces the expression of various antioxidant genes, such as HO-1, GCLC, GCLM, GST, and CAT, to mitigate oxidative damage, initially exerting tumor-suppressive effects [154,155]. During tumor progression, NRF2 transcriptionally regulates genes involved in proliferation, such as NOTCH1, BMPR1A, IGF1, ITGB2, JAG1, NPNT, PDGFC, and NQO1 [[156], [157], [158], [159]]. Additionally, NRF2 enhances immune evasion by upregulating PD-L1 and metabolic genes like SLC2A1, SLC7A11, and NAMPT, thereby impairing T cell-mediated cytotoxicity and promoting immunosuppression [160,161].

***p53 signaling.*** p53 is a transcription factor that, together with MDM2, forms the central hub of its signaling pathway, responding to environmental stress signals and mediating tumor suppression [162]. In a normal cellular environment, the E3 ubiquitin-protein ligase MDM2 directly binds to the N-terminal transactivation domain of p53, inhibiting its transcriptional activity and targeting it for proteasomal degradation via ubiquitination. Furthermore, p53 transactivates MDM2, which in turn downregulates p53, forming a negative feedback loop that limits excessive p53 accumulation and activity [163,164]. Upon receiving stress signals, MDM2 activity or its binding with p53 is inhibited, resulting in an extended half-life and nuclear accumulation of p53, leading to its transcriptional activation [165]. Among these stress signals, p53 is rapidly activated in response to DNA damage induced by ROS. This process mainly involves three members of the phosphatidylinositol 3-kinase-like kinase (PIKK) family: ATM, ATR, and the DNA-PK complex, which directly or indirectly phosphorylate p53 at specific serine residues, preventing its interaction with MDM2, and acetyl transferases such as CREBBP and KAT5, which activate p53 for specific DNA binding by acetylation [166,167]. p53 can also be directly modified by ROS through the oxidation of cysteine residues, thereby altering its conformation and function and promoting its DNA-binding ability [168] (Fig. 3b).

The classic tumor-suppressive functions of activated p53 are primarily manifested in its regulation of cell cycle arrest, apoptosis, and senescence through transcriptional control [165,169]. Specifically, p53 upregulates CDKN1A, which arrests the cell cycle to provide sufficient time for DNA repair. If the damage is irreparable, p53 induces apoptosis by upregulating pro-apoptotic genes such as BBC3, BAX, PUMA, and NOXA, while also promoting senescence by upregulating PML to inhibit proliferation [[170], [171], [172], [173], [174]]. Ultimately, these processes clear damaged cells from the tissue. However, approximately 50 % of human tumors harbor missense mutations in the p53 gene [175]. Interestingly, these mutations transform p53 from a tumor suppressor into a guardian of cancer, supporting the survival and progression of tumor cells [176]. This transformation is likely a result of selective pressure during tumor evolution and could provide insights into new therapeutic strategies.

***Hypoxia signaling.*** In tumors, hypoxia signaling is typically activated to cope with hypoxic stress, promoting tumor progression [177]. Under normoxic conditions, the two proline residues of HIF-α are hydroxylated by the proline hydroxylase family (PHDs), enabling interaction with von Hippel-Lindau protein (pVHL), which mediates the ubiquitination and subsequent proteasomal degradation of HIF-α [178,179]. However, under hypoxic conditions, PHD activity is inhibited, preventing HIF-α degradation. As a result, HIF-α dimerizes with HIF-1β, then translocating to the nucleus, where it binds to hypoxia-responsive elements to activate transcription and induce the expression of downstream genes [179,180].

HIF-α is also stabilized by ROS through the inhibition of PHDs, thereby further activating the hypoxia pathway [181]. In the hypoxic tumor microenvironment, activated HIF-1α inhibits c-Myc by upregulating the transcription of MAX interactor 1, thereby reducing the oxygen demand of tumor cells and shifting metabolism towards glycolysis [182]. Additionally, HIF-1α promotes angiogenesis by activating VEGF transcription and induces EMT through the transcriptional activation of Twist and Snail [183,184] (Fig. 3c). HIF-1α is also downstream of several previously mentioned pathways, working synergistically to promote tumor progression. This complex metabolic regulation highlights the adaptability and flexibility of cancer cells in different environments, suggesting that targeting a single point in the hypoxia pathway could inhibit multiple pro-tumor processes simultaneously, offering promising avenues for developing novel therapeutic strategies.

***FOXO signaling.*** FOXO signaling responses to stress conditions and regulate various physiological processes, including the cell cycle, metabolism, and antioxidant defense [185,186]. Generally, FOXOs are inactivated and retained in the cytoplasm due to negative regulation by the conserved PI3K/AKT signaling pathway. In the presence of insulin and growth factors, insulin receptors are activated, initiating a phosphorylation cascade sequentially involving IRS, PI3K, PDK1, and AKT, which leads to the phosphorylation of FOXOs. This phosphorylation increases FOXO binding to the chaperone protein 14-3-3, resulting in a conformational change that masks the nuclear localization signal and exposes the nuclear export signal, ultimately reducing FOXO nuclear localization and inhibiting its transcriptional activity [185,187]. Under oxidative stress, FOXO translocation to the nucleus is significantly increased through JNK signaling [188]. Additionally, JNK inhibits PI3K/AKT signaling at multiple levels by reducing IRS activity and dissociating FOXO from 14-3-3, further promoting FOXO-mediated transcription [189,190]. FOXO is also directly regulated by redox signals, forming heterodimers with transportin (TNPO) and acetyltransferase p300 via disulfide bridges, which enhances its nuclear translocation [191,192]. However, H_2_O_2_ can oxidize and inactivate the phosphatases PTP1B and PTEN, which typically inhibit PI3K/AKT signaling, thereby inhibiting FOXO transcriptional activity [193]. Other pathway components, including insulin receptors and AKT, are also subject to oxidative regulation by H_2_O_2_, leading to the inhibition of FOXO activity [194] (Fig. 3d).

FOXO signaling plays a paradoxical role in cancer progression [195]. While FOXOs are generally considered tumor suppressors, the PI3K/AKT pathway is often overactivated in many cancers, leading to FOXO inhibition and the suppression of its antitumor functions [196,197]. Paradoxically, in the highly oxidative environment of tumor cells, FOXO activity and nuclear localization can increase, promoting the transcription of antioxidant genes and reducing ROS levels by inhibiting hypoxia signaling, thereby mitigating ROS-induced pro-tumor effects [198]. FOXOs also exhibit overall inhibitory effects on various aspects of cancer, including metabolism, proliferation, and survival. Specifically, FOXO1 can suppress classic aerobic glycolysis (metabolic pattern required for tumor growth [199]), in part by inhibiting c-Myc, a transcription factor that is frequently upregulated in most cancers and drives cell cycle progression and anabolic metabolism [200,201]. Moreover, FOXOs downregulate lipogenesis and promote lipolysis by reducing the transcriptional activity of sterol regulatory element-binding protein 1 (SREBP1), a key regulator of lipid biosynthesis, while upregulating the expression of adipose triacylglycerol lipase (ATGL), a key enzyme in lipolysis [202,203]. FOXOs also induce cell cycle arrest by inhibiting the expression of cyclins and c-Myc [195,204]. However, recent research suggests that FOXOs may also support cancer progression [205]. FOXOs contribute to the maintenance of tumor stem cells, mediate drug resistance through the upregulation of the MDR1 protein, and enhance tumor invasiveness by increasing the expression of matrix metalloproteinases (MMP-9 and MMP-13) [195,206,207]. Notably, FOXOs exhibit a dual role in angiogenesis. Most studies suggest that FOXOs inhibit angiogenesis by suppressing endothelial cell proliferation via c-Myc inhibition and by downregulating HIF-1α, which reduces VEGF transcriptional activation [200,207]. However, the expression of VEGF-responsive genes, such as vascular cell adhesion molecule-1 (VCAM-1), depends on FOXO, thus also showing some promotive effects on angiogenesis [208]. Further research is needed to determine the balance between these opposing functions and to clarify the dominant role of FOXOs in different tumor types and context.

***NF-κB signaling.*** NF-κB represents another class of transcription factors regulated by oxidative stress, mainly mediates inflammatory responses [209,210]. Under resting conditions, NF-κB is controlled by the inhibitor of κB (IκB) family, which masks the nuclear localization signal (NLS) of NF-κB, preventing its transport into the nucleus [211]. NF-κB activation occurs through two primary signaling pathways: the canonical pathway, activated by most physiological stimuli, and the noncanonical pathway, triggered by certain TNF cytokines [212]. In both pathways, a common critical node is the activation of the IκB kinase (IKK) complex via intracellular signaling cascades. Once activated, IKK phosphorylates IκB and p100, marking them for polyubiquitination and subsequent degradation via the proteasome system. The degradation of IκB and p100 releases NF-κB, allowing it to translocate to the nucleus and function as a transcriptional activator [181].

NF-κB signaling is subject to dual regulation by ROS [213]. NF-κB activation can be promoted by ROS through the activation of upstream IKK, either via the direct modification of critical cysteine residues in IKK or through the ATM and ATR kinases, which phosphorylate IKKγ in response to oxidative DNA damage [214]. NF-κB activation is also enhanced by ROS through the serine and tyrosine phosphorylation of IκB [215,216]. However, NF-κB signaling can also be inhibited. For instance, KEAP1 induces H_2_O_2_-dependent degradation of IKKβ and reduces the DNA-binding capacity of the NF-κB complex by S-glutathionylation of Cys62 in the p50 subunit within the nucleus [217,218] (Fig. 3e). In many types of cancer, NF-κB is constitutively activated, often due to mutations in NF-κB coding genes or the continuous release of cytokines by macrophages in the TME, leading to its pro-tumorigenic effects [219]. Specifically, NF-κB promotes tumorigenesis by upregulating genes involved in cell cycle regulation (e.g., cyclin G1, cyclin D1) and angiogenesis (e.g., MCP-1, CXCL8, EGFR, EGFR receptors) [[220], [221], [222], [223], [224]]. Furthermore, NF-κB enhances tumor migration and metastasis by inducing the expression of genes such as Snail, Slug, Twist, ZEB, vimentin, and MMPs, which disrupt intercellular junctions, maintain the mesenchymal phenotype, and degrade the extracellular matrix, respectively [181]. Notably, NF-κB signaling has complex crosstalk with other pathways, coordinating responses to stress. For instance, there is mutual regulation between NF-κB and NRF2, as well as NF-κB's role in regulating HIF-1α in hypoxia signaling [225,226].

***MAPK/ERK pathway.*** The MAPK/ERK pathway is a central intracellular signaling cascade that transmits extracellular signals through a series of kinase reactions, regulating cell proliferation, differentiation, survival and is often persistently activated in tumor cells due to mutations in key genes [227]. Normally, it can be activated via the classical ligand-dependent pathway, where extracellular signal proteins (e.g., growth factors) bind to receptor tyrosine kinases (RTKs) located on the cell membrane, leading to RTK dimerization and autophosphorylation. This activates the RTK-Grb2-SOS signaling axis, initiating a three-tiered phosphorylation cascade that sequentially activates RAS, Raf1, and MEK1/2, ultimately resulting in the activation of the effector kinase ERK1/2 [228]. The MAPK/ERK pathway can be activated either directly or indirectly by ROS modifying key proteins. Mechanistically, the activation of the MAPK/ERK pathway involves oxidative modifications of the aforementioned pathway proteins, promoting their phosphorylation and thereby directly activating the pathway. The MAPK/ERK pathway can also be indirectly enhanced through the oxidative deactivation of certain protein phosphatases, such as PTEN and PTP, which maintains sustained phosphorylation of proteins within the pathway [229,230] (Fig. 3f).

Once activated, ERK1/2 translocates into the nucleus, where it phosphorylates numerous substrates [231]. Key cancer-related substrates include the proto-oncogene c-Myc, HIF1α (which promotes angiogenesis and migration), c-Fos (which activates CCND1 transcription to produce cyclin D1), and matrix metalloproteinases (MMPs) that facilitate invasion [[232], [233], [234], [235], [236]]. Thus, the MAPK/ERK pathway plays a crucial and comprehensive role in tumor promotion. While the widespread and highly frequent mutations in key pathway genes are the primary drivers of its persistent activation in tumor cells, the high levels of ROS present in the tumor microenvironment also contribute to the enhancement of this pathway. Therefore, targeting ROS-mediated amplification of MAPK/ERK signaling may present a potential anti-tumor strategy.

## 4 Oxidative stress and regulated cell death

When ROS levels exceed a certain threshold, excessive oxidative damage can become lethal, triggering various forms of RCD, including apoptosis, ferroptosis, cuproptosis, pyroptosis, necroptosis, parthanatos, NETotic cell death, paraptosis, lysosome-dependent cell death, and oxeiptosis. Here, we will explore the intricate regulatory interactions between ROS and RCD, as well as their potential applications in cancer therapy. It is also important to highlight that ROS-induced RCD can facilitate effective cancer therapy by eliminating tumor cells, and in some cases, ROS-induced RCD may paradoxically promote tumor progression and contribute to therapy resistance [11,237,238].

### 4.1 ROS and apoptosis

Apoptosis is a non-lytic, immunologically silent form of RCD driven by the activation of the caspase cascade, a group of cysteine proteases responsible for the degradation of subcellular components, including nuclear DNA, proteins, and cytoskeletal elements. This process culminates in the formation of apoptotic bodies, which are plasma membrane-bound vesicles containing cytoplasm with densely packed organelles and sometimes nuclear fragments, ultimately leading to phagocytosis by phagocytes [239,240]. As early as 1991, research identified ROS as a mediator of apoptosis within the blastocysts, associated with glutathione-dependent protective mechanisms [241]. Recent studies have further elucidated the role of ROS in inducing apoptosis across various biological systems, sometimes mediating therapeutic effects. For instance, in fungal systems, ROS disrupt mitochondrial function in *Candida albicans* upon itraconazole treatment, inducing apoptosis [242]. Similarly, in human gastric cancer SGC7901 cells, ROS modulate apoptosis-related proteins, thereby inhibiting cell proliferation and survival in response to Betulin [243]. Moreover, in primary lung epithelial cells, ROS are required for FasL-induced apoptosis [244].

ROS-induced apoptosis is initiated by extensive damage to intracellular biomacromolecules, with a particular impact on DNA [245,246]. mtDNA, being especially vulnerable to oxidative stress, suffers damage that contributes to mitochondrial dysfunction [240]. This damage impairs the mitochondrial ETC, leading to increased ROS production and establishing a self-amplifying cycle of ROS accumulation and mtDNA damage, which exacerbates apoptotic signaling [247]. The resulting ETC dysfunction causes loss of mitochondrial transmembrane potential and impairs inner membrane integrity, further activating apoptosis by promoting the irreversible opening of the mitochondrial permeability transition pore [248]. This pore opening increases non-specific membrane permeability, allowing the leakage of pro-apoptotic proteins such as cytochrome *c*, which subsequently initiates the caspase cascade and triggers cell death [249,250].

**Fig. 4:**
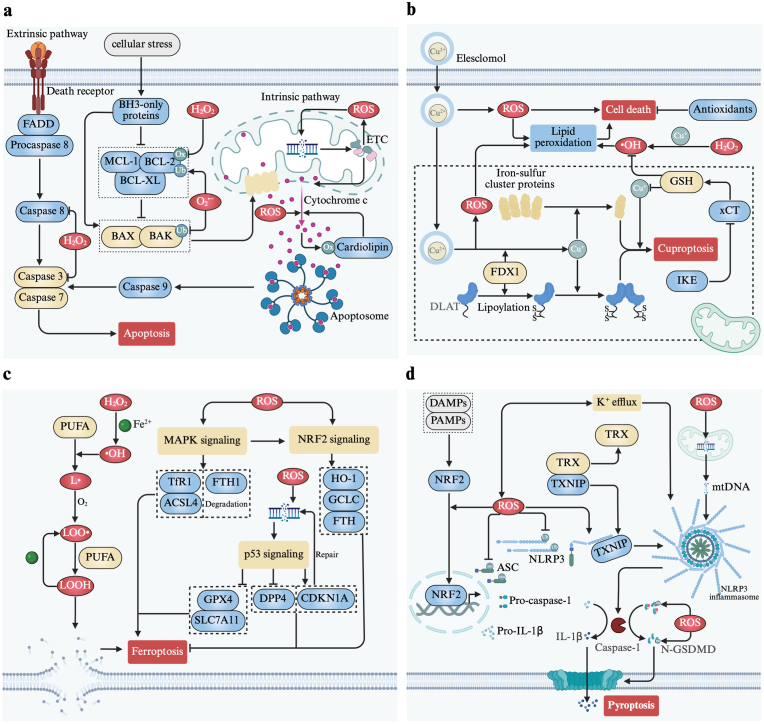
ROS-mediated regulated cell death. **a** Apoptosis is induced by ROS through damaging mitochondrial DNA, modifying apoptosis-related proteins, promoting cytochrome *c* release. **b** ROS are actively involved in the process of cuproptosis and induce cell death independent of FDX1. **c** ROS-induced lipid peroxidation leads to ferroptosis, and ROS modulate ferroptosis through various signaling pathways. **d** ROS participate in the priming and activation stages of inflammasome activation and activate gasdermin-mediated membrane rupture. **e** Necroptosis relies on TNF signaling and ROS-driven RIP1/RIP3 activation, leading to MLKL-dependent plasma membrane rupture. **f** Paraptosis is triggered by ROS-induced ER stress and mitochondrial swelling. **g** Lysosome-dependent cell death involves ROS-mediated LMP, releasing cathepsins that lead to cell death. **h** ROS activate and promote release of NE and MPO to facilitating NETs formation. **i** High ROS induce oxeiptosis through the KEAP1-PGAM5-AIFM1 pathway. **j** Parthanatos results from ROS-induced DNA damage, leading to PARP-1 overactivation and AIF-mediated DNA fragmentation.

ROS also influence apoptosis by modifying apoptosis-related proteins. At low mitochondrial ROS levels, cytochrome *c* increases its peroxidase activity, oxidizing cardiolipin, which facilitates cytochrome *c*'s detachment and translocation to the cytosol—a key step in the activation of the apoptosis pathway [251]. ROS further modulate the activity of Bcl-2 family proteins [252], where H_2_O_2_, for instance, oxidizes Bcl-2 at specific cysteine residues (Cys158 and Cys229) [253], and O_2_^•−^ influences the ubiquitination of proteins such as Bax, Bak, and Bcl-2 [254] (Fig. 4a). Interestingly, H_2_O_2_ can also reversibly inhibit caspases 3 and 8 by oxidizing catalytic cysteine sites, though ROS may simultaneously activate these caspases through upstream signaling events [254].

Considering tumor cells, often exposed to high ROS levels, display increased sensitivity to further ROS elevation [255]. Therefore, leveraging oxidative stress inducers to selectively provoke apoptosis in tumor cells has emerged as a promising therapeutic approach, offering targeted cytotoxicity against cancer cells [[255], [256], [257]].

### 4.2 ROS and cuproptosis

Cuproptosis is a newly identified form of RCD that is copper- and proteotoxic stress-dependent [127]. It was formally introduced by Tsvetkov et al., in 2022 to describe a distinct RCD mechanism, characterized by the oligomerization of lipidated proteins in the TCA cycle and the subsequent loss of iron-sulfur cluster proteins [258]. In 2012, Nagai et al. reported that elesclomol can continuously and selectively transport copper to mitochondria in the form of the elesclomol-Cu(II) complex, where Cu(II) is reduced to Cu(I) by the enzyme ferredoxin 1 (FDX1), generating significant amounts of ROS to induce cell death [259,260]. This copper-induced cell death lacks the hallmarks of apoptosis [259,261], was not well-understood until the formal recognition the concept of cuproptosis with the identification of mitochondrial FDX1 as a key regulator [258,262]. However, current research has not established a direct influence of ROS on FDX1-dependent cuproptosis, despite some sporadic reports. For example, copper ions can produce •OH through Fenton-like reactions, and mitochondrial copper transport enhances ROS production [263,264]. Gao et al. found that IKE can synergize with elesclomol-Cu to amplify cuproptosis by targeting xCT and FDX1, leading to mitochondrial damage, increased ROS levels, and GSH depletion [265]. GSH, a vital endogenous copper chelator as well as cofactor for GPX4, mitigates oxidative stress; its depletion leads to ROS accumulation and promote cuproptosis [263,266] (Fig. 4b). Li et al. showed that inhibiting the xCT-GSH-GPX4 pathway intensified disulfiram/Cu-induced cuproptosis in myelodysplastic syndromes, highlighting the link between antioxidant capacity and cuproptosis susceptibility [267]. Additionally, alternative pathways independent of FDX1 have been observed. Gale et al. demonstrated that elesclomol-Cu-induced cell death in astrocytes occurs via ROS production and lipid peroxidation, independent of mitochondrial respiration or FDX1, but can be mitigated by antioxidants [268].

Given the active involvement of ROS in the process of cuproptosis and their unavoidable substantial production, further research is needed to elucidate their exact influence on cuproptosis. Understanding this relationship is crucial, as tumor cells often exhibit elevated ROS levels [255], positioning cuproptosis as a potentially effective cancer therapy.

### 4.3 ROS and ferroptosis

Ferroptosis, a regulated form of cell death, is characterized by iron dependence and the lethal accumulation of membrane-localized specific lipid peroxides [269]. This process requires the oxidation of PUFA-containing lipids and often involves compromised cellular defenses against lipid peroxidation [270]. Central to the execution of ferroptosis are phospholipid hydroperoxides (PLOOHs), a lipid-based form of ROS [271]. Initially, lipid peroxides accumulate within organelles such as the endoplasmic reticulum and ultimately aggregate on the plasma membrane [272]. This accumulation disrupts ionic homeostasis through the activation of ion channels, leading to osmotic cell swelling and eventual plasma membrane rupture [273].

ROS play a pivotal role in modulating ferroptosis through various signaling pathways [274]. Elevated ROS levels can activate the MAPK signaling pathway, which induces ferroptosis by upregulating TfR1 and increasing ACSL4 expression while promoting the degradation of FTH1 [275]. However, the MAPK pathway also exerts protective effects by activating NRF2, which enhances the transcription of antioxidant and iron homeostasis genes, such as HO-1, GCLC, and FTH, to counteract oxidative stress [[275], [276], [277]]. Additionally, ROS-induced DNA damage can rapidly activate the p53 signaling pathway, which has a dual role in ferroptosis regulation [278]. On one hand, p53 promotes ferroptosis by repressing SLC7A11 and GPX4 expression [279,280], while on the other, it can inhibit ferroptosis by preventing DPP4 activity or enhancing CDKN1A expression to facilitate DNA repair and preserve GPX4 function [281,282] (Fig. 4c).

The high basal ROS levels inherent to many tumor cells further create a permissive environment for ferroptosis induction [283]. Therefore, understanding the intricate interactions between ROS, ferroptosis, and tumor biology holds significant potential for developing targeted therapies, particularly for aggressive, treatment-resistant cancers.

### 4.4 ROS and pyroptosis

Pyroptosis is a form of RCD executed by gasdermins, morphologically characterized by continuous cell swelling and the formation of large bubbles on the cell membrane until rupture [284,285]. The classical pathway of pyroptosis involves the activation of the inflammasome, a multiprotein complex comprising NLRs, ASC, and pro-caspase-1. This complex activates caspase-1, which cleaves gasdermin D (GSDMD) into an N-terminal pore-forming domain and a C-terminal repressor domain, while simultaneously processing pro-inflammatory cytokines IL-1β and IL-18 into their mature forms [286,287]. The N-terminal domain of GSDMD oligomerizes to form membrane pores, causing cell lysis and the release of inflammatory mediators [288].

The activation of the canonical NLRP3 inflammasome pathway typically requires two stages: priming and activation [289]. ROS serve as key upstream signals in both phases [290,291]. During the priming phase, ROS activate the MAPK pathway, leading to NF-κB activation and the subsequent transcriptional upregulation of NLRP3 and pro-IL-1β [292,293]. At the post-translational level, mitochondrial ROS, alongside Toll-like receptor signaling, promote the deubiquitination of NLRP3 at Lys63, facilitating its activation [292,294,295]. Additionally, ROS facilitate ASC deglutathionylation at Cys171, enhancing ASC oligomerization and subsequent NLRP3-ASC complex assembly [296]. In the activation phase, ROS act in concert with K^+^ efflux, a critical step for inflammasome assembly [297]. ROS also trigger the dissociation of thioredoxin-interacting protein (TXNIP) from TRX upon oxidation, allowing TXNIP to bind NLRP3 and promote inflammasome assembly [298]. Moreover, ROS-induced mitochondrial DNA (mtDNA) oxidation results in fragmented mtDNA release into the cytosol, further stimulating NLRP3 inflammasome assembly [299,300]. Upon full NLRP3 inflammasome assembly, pro-caspase-1 auto-activates into caspase-1, cleaving GSDMD to release the N-terminal pore-forming domain, which perforates the cell membrane and induces pyroptosis [284]. ROS can modulate GSDMD by oxidatively modifying specific cysteines (Cys38, Cys56, Cys268, Cys467 in humans, and Cys39, Cys57, Cys265, Cys487 in mice), enhancing caspase-1 cleavage efficiency [301]. ROS can also drive the S-palmitoylation of GSDMD's N-terminal domain by upregulating palmitoyl transferase expression to promote pore-forming activity [302,303] (Fig. 4d). Interestingly, ROS-dependent S-palmitoylation occurs in intact GSDMD, which can similarly form pores and mediate pyroptosis through liposome leakage [303].

Beyond the classical pathway, ROS can activate other gasdermin-dependent pyroptosis pathways. For example, in melanoma cells, iron-activated ROS trigger GSDME-dependent pyroptosis via the Tom20-Bax-caspase-9-caspase-3-GSDME axis [304]. In breast cancer, doxorubicin-induced ROS activate the JNK pathway or caspase-8, leading to caspase-3 activation and subsequent GSDME cleavage [305]. Additionally, α-ketoglutarate elevates ROS levels to induce GSDMC-dependent pyroptosis by oxidizing death receptor DR6, which recruits caspase-8 and GSDMC for activation [306]. Ultraviolet-C irradiation has also been found to induce full-length GSDME-mediated pyroptosis by promoting GSDME PARylation and subsequent lipid ROS-induced oligomerization [307].

The inherent high ROS levels in tumor cells present an opportunity to harness ROS-induced pyroptosis for cancer therapy [308,309]. Various compounds, such as lobaplatin and simvastatin, have been shown to trigger ROS-dependent pyroptosis, demonstrating potential anti-tumor effects [310,311]. However, pyroptosis is a double-edged sword. The release of IL-1β and IL-18 during pyroptosis can promote chronic inflammation, which may support tumor growth and progression [238,[312], [313], [314], [315]]. Therefore, while ROS-induced pyroptosis holds promise as a therapeutic strategy, careful consideration of the pro-tumorigenic potential of chronic inflammation is essential for developing effective cancer treatments.

### 4.5 ROS and necroptosis

Necroptosis, a caspase-independent RCD, serves as a backup when apoptosis is inhibited, typically activated through ligands binding to death receptors, such as TNF-α and its receptor TNFR1, which is the most well-studied subtype [316]. The central event in necroptosis is the prevention of caspase-8 activation, which allows RIP1 and RIP3 activation, leading to MLKL phosphorylation. This process forms pores in the plasma membrane, releasing DAMPs, causing cell swelling, membrane rupture, and eventual cell death [317,318].

**Fig. 5:**
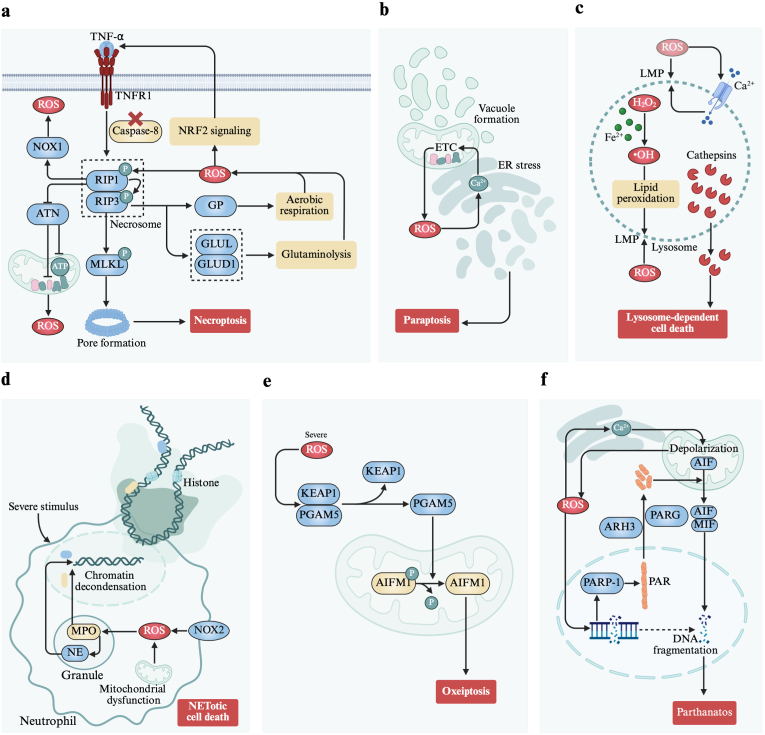
ROS-mediated regulated cell death. **a** Necroptosis relies on TNF signaling and ROS-driven RIP1/RIP3 activation, leading to MLKL-dependent plasma membrane rupture. **b** Paraptosis is triggered by ROS-induced ER stress and mitochondrial swelling. **c** Lysosome-dependent cell death involves ROS-mediated LMP, releasing cathepsins that lead to cell death. **d** ROS activate and promote release of NE and MPO to facilitating NETs formation. **e** High ROS induce oxeiptosis through the KEAP1-PGAM5-AIFM1 pathway. **f** Parthanatos results from ROS-induced DNA damage, leading to PARP-1 overactivation and AIF-mediated DNA fragmentation.

Studies have highlighted a close interplay between ROS and necroptosis, establishing a positive feedback loop. ROS can activate necroptosis, while key necroptotic components like RIP1 and RIP3 promote ROS generation, intensifying the cell death pathway [319]. Specifically, ROS production induced by various agents can initiate TNF-mediated necroptosis, reversible by ROS scavengers [[320], [321], [322]]. Mechanistically, ROS can activate the NF-κB signaling, upregulating TNF-α transcription, a necroptosis initiator [323]. Additionally, ROS target RIP1 at cysteine residues (Cys257, Cys268, and Cys586), forming disulfide bonds that induce RIP1 autophosphorylation at Ser161, which recruits RIP3 to form the necrosome [324]. Activated RIP1 and RIP3 further enhance ROS through metabolic pathways, creating a reinforcing feedback loop [324,325]. RIP1, through TNF-α, activates the NOX1 complex, promoting ROS production [326], and inhibits ANT, reducing ATP synthase activity and causing mitochondrial membrane potential hyperpolarization, thus increasing ROS levels [327,328]. RIP3 boosts ROS by upregulating enzymes like glycogen phosphorylase (GP), glutamate-ammonia ligase (GLUL) and glutamate dehydrogenase 1(GLUD1), enhancing aerobic respiration and glutaminolysis, both contributing to ROS production [319,329,330] (Fig. 5a).

Necroptosis requires caspase-8 inhibition, which also suppresses apoptosis. Interestingly, inhibiting caspases can enhance cell death immunogenicity, beneficial in cancer therapy [331]. Furthermore, recent studies suggest necroptosis can overcome resistance to apoptosis-inducing anti-cancer agents, highlighting its potential as a therapeutic strategy [332]. By leveraging the high ROS background in tumors, necroptosis inducers (caspase inhibitors) may enhance anti-cancer efficacy.

### 4.6 ROS and paraptosis

Paraptosis, a form of RCD, was first identified in 2000 by Sperandio et al. through activation by the insulin-like growth factor 1 receptor, characterized by extensive cytoplasmic vacuolation due to the progressive dilation of the ER and mitochondria [333].

ROS playing a pivotal role in its initiation and progression. ROS are also continuously produced and accumulated, leading to sustained cellular stress and eventual cell death [130]. Specifically, excessive ROS disrupt protein folding and calcium balance in the ER, triggering ER stress and subsequent dilation [129]. Calcium released from the ER is taken up by the mitochondria via the mitochondrial calcium uniporter, leading to mitochondrial calcium overload, swelling, and further ROS production due to ETC disruption [334] (Fig. 5b).

In glioblastoma multiforme, Zhao et al. revealed that nitrovin exhibits antitumor activity by targeting thioredoxin reductase 1, which promotes ROS accumulation and induces paraptosis [335]. Additionally, anticancer agents like dimethoxycurcumin and curcumin have been shown to elevate ROS levels to induce paraptosis effectively [336,337]. Therefore, leveraging ROS-induced paraptosis has emerged as a promising strategy in cancer therapy, offering potential for novel anticancer agents designed to amplify oxidative stress.

### 4.7 ROS and lysosome-dependent cell death

Lysosome-dependent cell death (LDCD) is a form of RCD triggered by lysosomal membrane permeabilization (LMP), leading to the release of lysosomal contents like cathepsins, which act as primary executors of this pathway [10,338]. Among the mechanisms that induce LDCD, ROS-mediated lysosomal destabilization is particularly well-studied [131]. Due to a lack of effective antioxidant defenses, lysosomes are highly sensitive to ROS, making their membranes especially vulnerable to ROS-induced damage [131]. Additionally, lysosomes contain rich free iron, which catalyzes the formation of •OH that further damage the lysosomal membrane through lipid peroxidation [339,340]. ROS can also activate lysosomal calcium channels, contributing to LMP [341] (Fig. 5c).

In breast cancer cells, hexamethylene amiloride has been shown to induce LDCD by promoting ROS production, thereby demonstrating anti-tumor activity [342]. Given the elevated ROS levels typically found in tumor cells, further ROS induction to trigger LDCD presents a promising therapeutic strategy for cancer treatment.

### 4.8 ROS and NETotic cell death

NETotic cell death is a specialized form of RCD occurring in hematopoietic cells, particularly neutrophils [10,338]. This process is characterized by the extrusion of a decondensed DNA-based meshwork bound with histones, neutrophil elastase (NE), and myeloperoxidase (MPO), forming neutrophil extracellular traps (NETs). These NETs play a critical role in combating large, non-phagocytosable pathogens [10,343].

ROS production, largely via NOX2 activation or mitochondrial dysfunction, is essential for NETotic cell death and is triggered by increased intracellular calcium following cellular stimulation [344,345]. Specifically, ROS activate NE through the MPO pathway and facilitate the release of NE and MPO from neutrophil granules into the cytosol [346,347]. These proteins then translocate to the nucleus, where they drive chromatin decondensation, a hallmark of NETotic cell death [347] (Fig. 5d).

Unlike other cell death forms that primarily target tumor cells, NETotic cell death contributes to tumor progression by trapping tumor cells, which can promote metastasis [237]. Thus, inhibiting NETosis cell death may offer a novel therapeutic strategy in cancer treatment.

### 4.9 ROS and oxeiptosis

Oxeiptosis is an apoptosis-like form of RCD induced by elevated oxidative stress, mediated through the KEAP1-PGAM5-AIFM1 pathway, with dephosphorylation of AIFM1 at serine 116 (S116) as a key indicator [132]. It plays a significant role in counteracting ROS-induced inflammation [348] (Fig. 5e). The role of oxeiptosis in tumor biology remains largely unexplored. However, due to the high ROS levels typically present in tumor cells, it is plausible that these cells may be more susceptible to oxeiptosis when exposed to ROS-inducing agents.

### 4.10 ROS and parthanatos

Parthanatos, a type of RCD, is triggered by extensive and prolonged DNA damage, leading to the overactivation of the nuclear DNA repair enzyme PARP-1. This overactivation results in chromatin condensation and DNA fragmentation [349]. PARP-1 synthesizes PAR polymers to mark DNA damage, which are subsequently cleaved by PARG and ARH3 enzymes, enabling PAR to exit the nucleus and translocate to the mitochondria [350]. In the mitochondria, PAR induces the release of AIF, which binds with MIF to form a complex that returns to the nucleus to mediate DNA fragmentation, culminating in cell death [350].

Oxidative stress acts as the primary driver of the DNA damage that initiates parthanatos [129,351]. Cytosolic ROS can also trigger calcium release from the ER into the mitochondria, leading to mitochondrial depolarization and an increase in mitochondrial ROS, which exacerbates DNA damage and further promotes parthanatos [352] (Fig. 5f). Tumors, characterized by high ROS levels, experience enhanced DNA damage [7], making tumor cells more susceptible to parthanatos than normal tissues.

## 5 Oxidative stress-mediated regulated cell death in cancer therapy

Tumor cells, due to their higher basal ROS levels compared to normal tissues, are more vulnerable to oxidative stress-induced cell death [255,353,354]. Traditional anti-tumor therapies, such as radiotherapy and chemotherapy, partly leverage this vulnerability by increasing ROS levels, although this is not their primary mechanism of action. Photodynamic therapy, a more targeted approach, induces ROS through photochemical reactions, leading to cancer cell death via pathways like apoptosis, paraptosis, and necroptosis [353,354].

These therapeutic strategies, however, often have broad effects and may disrupt normal physiological processes. Achieving selective tumor cell death while minimizing damage to normal tissues remains a key challenge [129]. Here, we explore the potential of existing non-cancer drugs to selectively target redox mechanisms to modulate ROS levels and exert anti-tumor effects, despite these actions not being their primary pharmacological purpose. This perspective may reveal whether drugs approved for non-oncologic conditions, which can influence ROS production, might also have anti-tumor potential.

### 5.1 Targeting NOXs

NOXs are unique enzymes dedicated to ROS production [43,355]. In various cancers, NOXs overexpression is often driven by oncogenic transformations, such as RAS mutations and elevated pro-inflammatory cytokines, leading to increased ROS production and promoting tumor progression [[356], [357], [358]]. Given this, NOXs are considered promising drug targets for controlling ROS biology and inhibiting tumor development [359](Table 1).

**Table 1:** Agents that target NOXs in cancer therapy.

Compounds	Targets	Types of RCD	Cancer types	Key findings	Limitations	Phase
Diphenylene iodonium (DPI)	NOX	Apoptosis	Colon [364]	Target the FAD domain of NOXs Arrest cell cycle	Off-target effect: inhibit ETC, block CYP450 reductase, inhibit NO synthase	Preclinical
Apocynin	NOX	Apoptosis	Breast [371] Prostate [373]	Suppress NOX components expression Interfere NOXs assembly	NOX-inhibitory effects require MPO	Preclinical
Setanaxib	NOX1 NOX4	Apoptosis	Breast [377] SCCHN	Enhance low-dose radiotherapy Overcome CAF-mediated tumor immunotherapy resistance First NOXs inhibitor to enter clinical trial	Clinical trials are primarily focused on non-cancer diseases	Phase II (NCT05323656)
ML171	NOX1	Apoptosis	Colon [379]	Inhibit the formation of ECM-degrading invadopodia	Off-target effect: antagonistic activity on serotonin and dopamine receptors	Preclinical
GKT771	NOX1	Apoptosis	Liver [381] Colon [380]	High specificity Anti-inflammatory Anti-fibrosis Inhibit angiogenesis Recruite immune cell	Not enter clinical trials and compete with Setanaxib	Preclinical
GSK2795039	NOX2	Apoptosis	N/A	The first small molecule inhibits NOX2 in vivo Inhibit the utilization of NADPH and oxygen	Lack of application in tumors	Preclinical

***Diphenylene iodonium (DPI) and di-2-thienyliodonium (DTI).*** DPI and DTI, both iodonium analogs, as inhibitors of general flavoprotein, are also classical non-specific and non-isoform selective NOX inhibitors [[360], [361], [362]]. By binding to the FAD domain, they block electron transfer, reducing NOX activity and mRNA expression [361,362]. Research has confirmed that in human colon cancers, DPI and DTI inhibit tumor growth through cell cycle arrest [362] but disrupt normal cellular functions due to off-target effects, such as mitochondrial complex I inhibition [361,[363], [364], [365]]. Consequently, they have not advanced to clinical trials, underscoring the need for more selective NOX-targeted drugs.

***Apocynin.*** Apocynin is a phenolic compound that selectively inhibits NOX by suppressing the expression of NOX components and blocking the assembly of NOX components [361,[366], [367], [368]]. Apocynin has shown anti-invasive effects in breast cancer models [369]. However, it requires myeloperoxidase (MPO) activation to exert NOX inhibition, limiting its efficacy in MPO-deficient tissues [368,370]. Furthermore, Suzuki et al. demonstrated that in prostate cancer, apocynin exerts antiproliferative effects through the dephosphorylation of Rac1 and the downregulation of cyclin D1, rather than directly targeting NOXs [371]. Therefore, apocynin remains a preclinical agent, as it lacks specific NOX-inhibitory properties in various biological systems.

***Setanaxib.*** A selective NOX1 and NOX4 inhibitor, setanaxib is the first NOX inhibitor to enter clinical development [[372], [373], [374]]. In breast cancer models, it enhances anti-cancer effects when combined with low-dose radiotherapy [375]. And in cancer-associated fibroblast (CAF)-rich tumors, such as breast cancer, lung cancer and colorectal cancer, setanaxib more effectively counteracts CAF-mediated immunosuppression than TGFβ1 inhibition, which is a key regulator of the CAF phenotype [376]. Although primarily under investigation for non-cancer conditions like liver fibrosis, its potential in oncology warrants further exploration.

***2- Acetylphenothiazine (ML171).*** ML171, a phenothiazine compound, selectively inhibits NOX1, showing anti-invasive effects in colon cancer by blocking ECM-degrading invadopodia [377]. However, due to its structural similarity to serotonin and dopamine receptor antagonists, ML171 may cause off-target effects, though this also facilitates its pharmacokinetic evaluation for potential in vivo applications [360].

***GKT771.*** GKT771, a highly specific NOX1 inhibitor with minimal off-target effects, demonstrates anti-tumor activity in liver and colorectal cancer models [[378], [379], [380], [381]]. By inhibiting angiogenesis and modulating the immune response, GKT771 enhances anti-tumor effects in colon cancer and hepatocellular carcinoma, especially when combined with anti-PD1 antibodies [378,379]. However, its specificity for a single NOX isoform may limit its applicability in cancers involving multiple NOX isoforms.

***GSK2795039.*** GSK2795039, a 7-azaindole molecule, is the first selective NOX2 inhibitor shown to function in vivo [382]. GSK2795039 competitively inhibits NADPH and oxygen utilization by NOX2 [382]. Although its role in oncology is unexplored, its high specificity suggests potential for treating NOX2-driven tumors.

### 5.2 Targeting the antioxidant enzymes

**GPX4 inhibitors.** GPX4, a critical regulator of ferroptosis, primarily functions by reducing lipid hydroperoxides to lipid alcohols [383,384]. Targeting GPX4 to induce ferroptosis is a promising strategy for overcoming acquired drug resistance, as persister tumor cells rely on GPX4 for survival [385]. This approach has shown therapeutic efficacy in eliminating cancer cells [386](Table 2).

**Table 2:** Agents that target antioxidant enzymes in cancer therapy.

Compounds	Targets	Types of RCD	Cancer types	Primary pharmacological actions	Limitations	Phase
GPX4 inhibitors						
Altretamine	GPX4	Ferroptosis	HIV-Related Cancer	Antineoplastic drug used in the treatment of advanced or recurrent ovarian cancer	Precise mechanism of inhibiting GPX4 in cancer treatment remains unclear.	Phase I (NCT00002936)
Withaferin A	GPX4	Ferroptosis	Neuroblastoma [394] Liver cancer [396] Ovarian cancer	Ferroptosis inducer	N/A	Phase I and II (NCT05610735)
Statins	GPX4	Ferroptosis	Triple-negative breast cancer [399]	Lipid-lowering drugs	Not all statins possess the same ability to induce ferroptosis	N/A
Acetaminophen	GPX4	Ferroptosis	N/A	Analgesic and antipyretic drug	Induction of ferroptosis depends on CYP2E1 expression	N/A
Ammonium Ferric Citrate	GPX4	Ferroptosis	Non-small-cell lung carcinoma [405]	Food additive for iron fortification to treat iron-deficiency anemia	N/A	N/A
**TXNRD inhibitors**						
Auranofin	TXNRD	Paraptosis Ferroptosis	Breast cancer [412] Lung neuroendocrine tumor [413] Small cell lung cancer [413] Ovarian Cancer Chronic lymphocytic leukemia Fallopian tube cancer	Treat rheumatoid arthritis	Weak selectivity	Phase II (NCT03456700) Phase II (NCT01419691) Phase I (NCT02126527) Early phase I (NCT01747798) Phase I and II (NCT01737502)
Mitomycin C	TXNRD	Apoptosis	Bladder cancer Urothelial carcinoma Anal carcinoma Ovarian cancer Upper tact urothelial carcinoma Breast cancer	Antibiotic chemotherapeutic drug Antifibrotic agent in surgical procedures	Antitumor effects targeting TXNRD is limited	Early phase I (NCT00734994) Phase II (NCT03658304) Early phase I (NCT01858025) Phase I (NCT05979909) Phase III (NCT01149174) Phase II (NCT01196455)
PX-12	TXNRD TXN	Apoptosis	Pancreatic cancer [423]	Anticancer drug	A phase II study of pancreatic cancer shows limited overall efficacy	Phase II (NCT00417287) Phase I (NCT00736372)
Ebselen	TXNRD	Apoptosis	Breast cancer [425]	Antioxidant and anti-inflammatory agent	Current research on the pharmacological effects of ebselen through TXNRD inhibition mainly focuses on combating bacterial or viral infections	N/A
Irofulven	TXNRD	Apoptosis	Renal cell carcinoma [433] Prostate cancer Colorectal cancer Melanoma Breast cancer Cervical cancer Pancreatic cancer Gastric cancer	Semi-synthetic anticancer drug	A phase II trial of metastatic renal cell carcinoma shows limited efficacy	Phase II (NCT00124566) Phase II (NCT00003441) Phase II (NCT00005968) Phase II (NCT00003796) Phase II (NCT00005070) Phase III (NCT00033735) Phase III (NCT00062257)
**SODs inhibitors**						
ATN-224	SOD1	Apoptosis	Breast cancer [439] Prostate cancer [439] Hormone-refractory [439] prostate cancer [439] Melanoma Breast cancer Hepatocellular carcinoma Esophageal carcinoma	Copper chelator	N/A	Phase II (NCT00383851) Phase II (NCT00405574) Phase I and II (NCT00352742) Phase II (NCT00674557) Phase II (NCT00150995) Phase II (NCT00006332) Phase II (NCT00176800)
Disulfiram	SOD1	Ferroptosis Apoptosis	Myeloma [442] Breast cancer Gastric cancer Glioblastoma Pancreatic Cancer Lung cancer	Treat chronic alcohol dependence	N/A	Phase II and III (NCT02678975) Phase I and II (NCT00256230) Early phase I (NCT03151772) Phase I (NCT02671890) Phase II (NCT03323346) Phase II and III (NCT00312819)
**CAT inhibitors**						
Arsenic trioxide	CAT	Apoptosis Ferroptosis	Glioma [378] Small cell lung cancer [496] Basal cell carcinoma Acute promyelocytic leukemia Myeloma Urothelial cancer	Treat acute promyelocytic leukemia	N/A	Phase I (NCT00003630) Phase II (NCT03624270) Phase II (NCT01470248) Phase II (NCT00017433) Phase II (NCT00009867)
BT-Br	CAT	Ferroptosis	Prostate cancer [366]	CAT inhibitor	N/A	Preclinical

***Altretamine.*** An FDA-approved drug for advanced ovarian cancer, altretamine's N-demethylation intermediates, hydroxymethylmelamines, are responsible for its cytotoxicity [387,388]. Recent studies suggest that altretamine inhibits GPX4, leading to ROS and lipid peroxide accumulation and triggering ferroptosis in tumor cells, although the precise mechanism remains unclear [389,390].

***Withaferin A.*** A natural compound derived from *Withania somnifera*, Withaferin A induces ferroptosis by targeting GPX4, reducing its expression and activity [391,392]. In neuroblastoma models, high doses of Withaferin A demonstrate a potent anti-tumor effect via the canonical ferroptosis pathway [392]. At moderate doses, it triggers a noncanonical pathway by activating KEAP1 and upregulating HO-1, increasing intracellular Fe (II) and promoting tumor cell death [393]. Withaferin A's potential is further enhanced through immune checkpoint inhibitor combinations in liver cancer [394], and nanoparticle formulations have improved its systemic delivery and tumor-targeted efficacy [392].

***Statins.*** Commonly used for lipid reduction, statins inhibit HMG-CoA reductase, which is the rate-limiting enzyme of the mevalonate pathway [395]. By impairing the maturation of tRNA^[Ser]Sec^, statins indirectly inhibit GPX4 synthesis, leading to lipid peroxidation and ferroptosis, particularly in triple-negative breast cancer [396,397]. Statins’ tumoricidal effect is synergistic when combined with direct GPX4 inhibitors, such as RSL3, especially in high-mesenchymal state cancer cells [398]. However, not all statins are equally effective in inducing ferroptosis, warranting further research [399].

***Acetaminophen.*** Known for its analgesic and antipyretic properties, acetaminophen is metabolized by cytochrome P450, family 2, subfamily E, polypeptide 1 (CYP2E1) into N-acetyl-*p*-benzoquinone imine (NAPQI), a reactive metabolite that depletes GSH and inhibits GPX4, inducing ferroptosis in primary hepatocytes [400]. However, NAPQI-induced ferroptosis does not occur in HepG2 hepatoma cells due to their inability to produce NAPQI [400,401]. Investigating this mechanism in other cancer cell lines with high CYP2E1 expression may reveal acetaminophen's potential in inducing ferroptosis.

***Ammonium Ferric Citrate.*** Commonly used as an iron supplement, ammonium ferric citrate has recently been shown to induce ferroptosis in non-small-cell lung carcinoma through the GPX4-GSS/GSR-GGT axis, offering a novel anti-tumor strategy [402,403].

**TXNRD inhibitors.** TXNRD, a selenoprotein, plays a crucial role in cellular defense against oxidative stress by using NADPH to reduce TXN, which in turn reduces target proteins via cysteine thiol-disulfide exchange [404]. In tumors, the overexpression of TXNRD helps counteract elevated ROS levels, thereby promoting angiogenesis and tumorigenesis [404,405]. This makes TXNRD a promising target for anticancer therapies [406](Table 2).

***Auranofin.*** Originally approved in the 1980s for rheumatoid arthritis, the gold-based compound Auranofin is now being repurposed for cancer treatment [407,408]. The gold ion (Au(I)) in Auranofin binds covalently to the selenocysteine residue at TXNRD's active site, inhibiting its function and exerting antitumor effects [409]. Recent studies indicate that 4–5 μM concentrations of Auranofin induce paraptosis in breast cancer cells by dual targeting TrxR1 and the proteasome [410]. Additionally, Auranofin enhances the sensitivity of lung neuroendocrine tumor cells to sorafenib by inhibiting TXNRD and has been shown to induce ferroptosis at higher doses [411,412]. However, its weak selectivity in targeting cancer cells presents a challenge for its clinical use [410].

***Mitomycin C.*** Known primarily as a DNA-alkylating chemotherapeutic agent, mitomycin C has shown TXNRD inhibitory activity as well [413,414]. Its enzymatic activation inside cells generates a highly reactive intermediate that forms interstrand DNA cross-links, contributing to its cytotoxicity [415,416]. Studies suggest that mitomycin C can alkylate TXNRD's active site, resulting in time- and concentration-dependent inhibition, which enhances its antitumor efficacy [417]. Nevertheless, since mitomycin C's primary mechanism centers on DNA damage, its utility as a TXNRD inhibitor remains underexplored.

***1- methylpropyl 2-imidazolyl disulfide (PX-12).*** PX-12 targets the thioredoxin system by oxidizing the catalytic cysteines in TXNRD and TXN, irreversibly inhibiting Trx and acting as a competitive substrate for TXNRD [404,418,419]. Phase I trials of PX-12 have shown acceptable tolerability in patients with advanced solid tumors [420], but a phase II trial in pancreatic cancer patients reported limited efficacy [421]. The anticancer potential of PX-12 thus warrants further investigation.

***Ebselen.*** Originally investigated as an antioxidant and anti-inflammatory agent, ebselen mimics glutathione peroxidase activity and has since been recognized as a TXNRD inhibitor [[422], [423], [424]]. In the MCF-7 human breast cancer animal model, ebselen can inhibit tumor growth by up to 50 % by targeting TXNRD [423]. Despite these promising results, most studies on ebselen's TXNRD inhibition focus on bacterial or viral infections, and further research is needed to validate its potential in cancer therapy [425,426].

***Irofulven.*** A semi-synthetic derivative of illudin S, irofulven acts primarily through DNA damage, leading to cell cycle arrest and apoptosis [427,428]. Additionally, irofulven binds to cysteine residues at TXNRD's active site, inhibiting its activity [429]. Although phase I trials have demonstrated its safety and preliminary efficacy in various solid tumors [430], phase II trials in metastatic renal cell carcinoma have shown limited success [431]. Continued research is essential to enhance irofulven's efficacy, particularly in understanding its mechanism of action through TXNRD inhibition.

**SODs inhibitors**. SODs catalyze the dismutation of O_2_^•−^ into H_2_O_2_ and O_2_, providing a critical first line of defense against ROS in most oxygen-dependent organisms [88]. Elevated SOD levels have been observed in various tumor tissues and are associated with poorer prognoses [432,433]. Inhibition of SOD2 in nasopharyngeal carcinoma, for example, has been shown to increase cancer cell sensitivity to ionizing radiation by inducing ferroptosis, highlighting SODs as potential prognostic markers and therapeutic targets [434](Table 2).

***Tetrathiomolybdate (ATN-224)*.** ATN-224, a copper-chelating small molecule, selectively inhibits SOD1 by binding intracellular copper, thereby reducing angiogenesis and promoting apoptosis [435,436]. Phase II trials have shown promising results in breast, prostate, and hormone-refractory prostate cancers, with ATN-224 exhibiting good safety, tolerability [437]. Ongoing studies are exploring combinations with other anticancer agents, with larger randomized trials anticipated to advance our understanding of its antitumor mechanisms, especially regarding metastasis [437].

***Disulfiram.*** Initially approved for treating alcohol dependence, disulfiram has recently demonstrated anticancer potential by inhibiting SOD1 [[438], [439], [440]]. In bortezomib-resistant multiple myeloma, disulfiram enhances the efficacy of bortezomib by targeting SOD1 [440]. Additionally, a combination of disulfiram and copper has shown promise in overcoming drug resistance in cancer therapy [441]. Given its low cost and favorable safety profile, disulfiram's anticancer applications merit further investigation, with a Phase II trial currently underway for metastatic breast cancer [441].

**CAT inhibitors**. CAT decomposes H_2_O_2_ into H_2_O and O_2_ and is often overexpressed in certain cancers, particularly acute myeloid leukemia [442,443]. In chronic lymphocytic leukemia and lymphoma, elevated CAT levels are associated with aggressive disease progression and chemoresistance [444,445]. Therefore, CAT could serve as a prognostic marker and a therapeutic target in cancer treatment (Table 2).

***Arsenic trioxide (ATO).*** Approved by the FDA for acute promyelocytic leukemia, ATO has demonstrated antitumor activity in other cancers, including glioma, small cell lung cancer, and basal cell carcinoma [[446], [447], [448]]. ATO reduces CAT activity by inhibiting CAT gene expression through the Akt pathway, which decreases FOXO transcription factor activity [449,450]. Additionally, ATO influences retinoic acid receptor α expression and directly suppresses CAT promoter activity, providing a multifaceted approach to lowering CAT expression [450]. These mechanisms make ATO a compelling candidate for targeting CAT-overexpressing tumors.

***BT-Br.*** BT-Br, a benzaldehyde thiosemicarbazone derivative, is the first synthetic CAT inhibitor that targets the NADPH-binding site, which is crucial for CAT activity preservation [451,452]. Preclinical studies in castration-resistant prostate cancer models have shown that BT-Br induces ferroptosis through CAT inhibition, resulting in notable antitumor effects [451,452]. However, BT-Br remains in the early stages of research, with further studies needed to optimize its mechanism and evaluate its clinical potential.

### 5.3 Targeting NRF2

The transcription factor NRF2 is the key mediator of the cellular antioxidant response [151]. In the premalignant stages of tumorigenesis, NRF2 exerts tumor-suppressive effects by upregulating the expression of antioxidant genes to mitigate oxidative damage, thereby preventing DNA damage and the accumulation of mutations [154,155]. Therefore, activating NRF2 is considered a potential therapeutic strategy, particularly in the early stages of cancer development (Table 3).

**Table 3:** Agents that target NRF2 in cancer therapy.

Compounds	Targets	Types of RCD	Cancer types	Key findings	Limitations	Phase
Isothiocyanate sulforaphane	NRF2	Apoptosis	Prostate [458,460] Breast [459,460] Colon [459]	Form a covalent bond with cysteine residues on Keap1 As a dietary supplement in early clinical trials for cancer prevention First identified NRF2 activators	Relatively low bioavailability Uncertain optimal dosage Limited therapeutic index	Phase II (NCT01265953)
Dimethyl fumarate	NRF2	Apoptosis	Large B-cell lymphoma [463] Chronic lymphocytic leukemia [464] Cutaneous T Cell Lymphoma	Pharmacological safety profile is well established Clinical trials have shown side effects to be transient	Multi-target: activate inflammatory pathways and immune regulation	Phase II (NCT 02546440)
Cyanoenone triterpenoids	NRF2	Apoptosis	Myeloma [470] Leukemia [470] Sarcoma [470] Lymphomas	The most potent NRF2 activators identified to date 300 synthetic derivatives with enhanced bioactivity have been developed	Intricate regulatory networks, including NF-κB, PTEN, and PI3K/Akt signaling pathways	Phase I (NCT00322140)

***Isothiocyanate sulforaphane.*** Sulforaphane, a naturally occurring compound derived from cruciferous vegetables, is one of the first identified NRF2 activators [453]. It exerts its effects by forming a covalent bond with cysteine residues on Keap1, inducing a conformational change that inhibits Keap1's negative regulation of NRF2 [454,455]. In breast cancer, colon cancer, and prostate cancer, sulforaphane has shown inhibitory effects on tumor growth and the promotion of tumor cell apoptosis [[454], [455], [456]] Currently, sulforaphane is being used as a dietary supplement in early clinical trials for cancer prevention. However, its relatively low bioavailability, uncertain optimal dosage, and limited therapeutic index hinder its clinical application as a therapeutic agent [454,457].

***Dimethyl fumarate (DMF).*** DMF was initially approved in 2013 for the treatment of multiple sclerosis [458]. However, recent research has increasingly focused on its potential as an anticancer agent, particularly due to its NRF2 agonist activity. This property has shown promise in treating hematological malignancies, including diffuse large B-cell lymphoma and chronic lymphocytic leukemia [459,460]. Currently, DMF remains in Phase II clinical trials as an anticancer agent due to the complexity of its multi-target mechanisms, including activation of inflammatory pathways and immune regulation [461,462]. Despite this, its pharmacological safety profile is well established, with common adverse effects such as gastrointestinal disturbances and flushing [463]. Clinical trials have shown these side effects to be transient, further supporting the potential of DMF as an adjunct in cancer therapy.

***Cyanoenone triterpenoids.*** Cyanoenone triterpenoids, derived from natural plants, represent the most potent NRF2 activators identified to date [464]. While naturally occurring cyanoenone triterpenoids often exhibit limited pharmacological activity, over 300 synthetic derivatives with enhanced bioactivity have been developed [465]. These compounds inhibit cancer cell proliferation by downregulating cyclin D1 and upregulating caveolin-1, while simultaneously activating both intrinsic and extrinsic apoptotic pathways to induce cell death, including common epithelial carcinomas, myeloma, leukemia, and sarcoma [464,466,467]. Currently, cyanoenone triterpenoids demonstrate greater therapeutic efficacy in early-stage tumors, and a Phase I clinical trial for solid tumors and lymphomas has been completed. However, due to their intricate regulatory networks—including NF-κB, PTEN, and PI3K/Akt signaling pathways—determining optimal dosages for cancer treatment remains a significant challenge [467].

## 6 Measurement of ROS and oxidative damage

When studying ROS in biological systems, it's crucial to recognize that "ROS" encompasses various molecules with distinct characteristics. This diversity, coupled with varying levels of expertise among researchers, often leads to inappropriate usage of ROS detection assays, potentially yielding misleading results [13]. This section provides an overview of well-established methods for detecting superoxide, hydrogen peroxide, and oxidative damage caused by hydroxyl radicals, such as lipid peroxidation, protein damage, and nucleic acid damage.

### 6.1 Superoxide detection

***SOD-inhibited cytochrome c reduction.*** The method is suitable for the quantitative detection of extracellular O_2_^•−^ but not intracellular O_2_^•−^ by utilizing its reductase activity toward cytochrome *c*^468^. The reaction is monitored spectrophotometrically at 550 nm, allowing for precise O_2_^•−^quantification, while SOD serves as a specificity control to distinguish genuine O_2_^•−^ activity from interference by other reducing agents [468]. It can be flexibly applied to both continuous and discontinuous multi-sample detection [469,470], though it is limited to extracellular measurements and may suffer interference from other redox-active molecules.

***Aconitase inactivation.*** This approach provides a qualitative measurement of mitochondrial O_2_^•−^ levels through monitoring the reversible inhibition of aconitase by O_2_^•−^-mediated [4Fe–4S] cluster disruption. Enzyme activity recovery upon O_2_^•−^ removal enables repeated measurements, making it suitable for long-term studies [[471], [472], [473]]. However, this method lacks absolute quantification and may be influenced by other ROS or iron perturbations.

***Dihydroethidium (HE) and MitoSOX Probes.*** These fluorescent probes enable intracellular and mitochondrial O_2_^•−^ detection, with the formation of 2-hydroxyethidium as a specific product [474]. While sensitive, these probes require careful optimization to avoid artifacts: liquid chromatography-mass spectrometry (LC-MS) is recommended to resolve fluorescence overlap [475], and low probe concentrations minimize quenching and auto-oxidation [476]. Additionally, mitochondrial membrane potential and morphology must be controlled to ensure accuracy [476].

### 6.2 Hydrogen peroxide detection

***Genetically encoded fluorescent probes***. HyPer and roGFP2-based genetically encoded probes provide highly sensitive and specific detection of cellular H_2_O_2_ through redox-dependent conformational changes [[477], [478], [479], [480]]. And their reversible nature makes them suitable for real-time H_2_O_2_ monitoring in live cells [477]. However, these probes require careful consideration of limitations including pH sensitivity (particularly HyPer) and the need for environment-specific calibration to ensure accurate interpretation [481,482].

***Boronate Probes.*** Upon oxidation by H_2_O_2_, boronate probes become fluorescent, allowing quantitative H_2_O_2_ measurement [[483], [484], [485]]. However, they may lack the sensitivity required for low physiological H_2_O_2_ concentrations [486]. And to ensure the probe's specificity towards H_2_O_2_, orthogonal methods or inhibitor controls can be employed to validate the specificity of the reaction [487,488].

***Amplex Red with HRP.*** This assay utilizes Amplex Red, which becomes fluorescent in the presence of H_2_O_2_ and horseradish peroxidase (HRP), providing a quantitative detection method, particularly for cell-released H_2_O_2_ [489,490]. The Amplex Reay be needed to control interference from other HRP substrates [490]. d-HRP system is highly sensitive, capable of detecting H_2_O_2_ at nanomolar (nM) concentrations [489].

### 6.3 Hypochlorous acid detection

***Fluorescent probe.*** Numerous commercially available fluorescent probes have been developed for the detection of hypochlorous acid (HOCl) both in vitro and in vivo [491]. These include mitochondria-targeted boron dipyrromethene probes for selective HOCl detection [492], ratiometric fluorescent naphthalimide probes capable of specific HOCl identification in complex environments [493], and colorimetric dual-channel probes characterized by low cytotoxicity and stability under physiological conditions [494]. Recently, a genetically encoded fluorescent probe, hypocrates, has been introduced, enabling reversible monitoring with a binding affinity in the range of 10^6^ M^−1^s^−1^ and providing dynamic real-time imaging in live cells and tissues [495]. However, challenges remain, as many probes exhibit limited selectivity, high costs, and suboptimal photophysical stability, necessitating further improvements for broader applications [491].

### 6.4 Singlet oxygen detection

***Singlet Oxygen Sensor Green (SOSG) fluorometric assay.*** This methodology exploits the ^1^O_2_-specific oxidation of the commercial SOSG probe, which undergoes structural isomerization to generate a fluorescent endoperoxide, resulting in signal amplification (the fluorescence color shifts from blue to green, Ex/Em: 504/525 nm) [496,497]. Quantitative assessment of ^1^O_2_ generation is achieved through fluorometric measurement of this stoichiometric conversion, with demonstrated specificity and nanomolar-level sensitivity [498]. And the technique exhibits effective in both in vitro cellular models (e.g., 3D tumor spheroids) and in vivo systems through intravital imaging of mammary tumors in Balb/c mice [499,500]. However, the SOSG probe cannot penetrate cells, limiting its ability to detect intracellular ^1^O_2_. To address this limitation, Hou et al. developed a new ratio-based probe, derived from the SOSG probe, to effectively monitor dynamic intracellular ^1^O_2_ changes [501].

### 6.5 Lipid peroxidation detection

***4-Hydroxynonenal (HNE) and Malondialdehyde (MDA).*** LC-MS/MS allows the most sensitive and specific quantification of HNE and MDA, with ELISA and immunohistochemistry providing additional options [[502], [503], [504], [505]]. ELISA is suitable for screening large numbers of samples and IHC offer insights into spatial distribution, though antibody specificity may vary and is susceptible to matrix effects and experimental manipulation [504,505].

***F2-Isoprostanes (F2-IsoPs).*** LC-MS/MS is the gold standard for F2-IsoPs detection due to its high sensitivity and specificity, which can detect F2-IsoPs at concentrations as low as picomolar levels and accurately differentiate and quantify various F2-IsoP isomers [506]. ELISA provides a faster, high-throughput option but may suffer from cross-reactivity [507,508].

***BODIPY and Cis-Parinaric Acid (PnA) Probes.*** These fluorescent probes detect lipid peroxidation by reacting with peroxyl radicals [509]. BODIPY's lipophilicity allows even cell membrane distribution and the simplicity of detective method making it suitable for real-time monitoring of lipid peroxidation in cells [510,511]. However, due to the slower reaction rate of BODIPY with peroxyl radicals compared to antioxidants, the fluorescence changes of BODIPY may not fully reflect the true inhibitory effects of antioxidants on lipid peroxidation [511]. PnA can be incorporated into different types of phospholipids, and using HPLC, it is possible to distinguish and detect the oxidation levels of different phospholipids [512]. However, PnA is prone to photobleaching, which may affect the stability of its fluorescence measurement [513].

### 6.6 Protein oxidative damage detection

Protein carbonylation, a common marker of oxidative damage, is often measured via the DNPH derivatization reaction, producing dinitrophenylhydrazone (DNP) adducts which is detected through spectrophotometry [514,515]. ELISA and immunoblotting utilize antibodies specific to DNP to identify and quantify DNP-modified proteins, offering relatively higher specificity and sensitivity [516]. However, since not all oxidative protein products contain carbonyl groups, these methods cannot detect all types of protein oxidation [517]. LC-MS offers broader detection capabilities for diverse oxidative modifications [518]. To achieve a more comprehensive assessment of oxidative protein damage, a combination of above methods is recommended.

### 6.7 Nucleic acid oxidative damage detection

The comet assay provides an overall assessment of DNA damage [519]. Liquid chromatography-tandem mass spectrometry (UPLC-MS/MS) can quantify specific markers like 8-oxo-7,8-dihydro-2′-deoxyguanosine in DNA or 8-oxo-guanosine in RNA, offering high sensitivity for evaluating oxidative stress impact on nucleic acids [520,521].

## 7 Conclusions and Perspectives

Targeting ROS-mediated RCD represents a promising therapeutic strategy in oncology, offering both new opportunities and significant challenges. Specifically, since cancer cells exhibit a lower threshold for ROS-induced cytotoxicity compared to normal cells, targeting ROS-mediated RCD offers selective tumor elimination [105] and may synergize with conventional therapies. For example, in drug-resistant cancers, ferroptosis susceptibility is heightened [278,522], making ROS modulation a promising strategy to eradicate these cells. Furthermore, combining ROS-inducing agents with existing therapies [523,524], such as chemotherapy, could both circumvent resistance and amplify overall antitumor efficacy, highlighting the translational potential of redox-based approaches. Additionally, the compensatory upregulation of antioxidant systems in tumors [525,526] provides additional druggable vulnerabilities. Despite the promise, several challenges remain to be addressed in future research.

Firstly, detecting cellular ROS levels present significant challenges. Although LC-MS offers high sensitivity and specificity for detecting most ROS types and oxidative damage [475,506,518,520,521], its complexity, high cost, and limited capacity for real-time monitoring hinder its broader adoption. Other methods, including HE and MitoSOX probes [475] for SODs detection, boronate probes [486] and Amplex Red [490] for H_2_O_2_ detection, ELISA [477] for lipid peroxidation detection, and immunoblotting [516] for protein oxidative damage detection, suffer from low specificity, limited sensitivity, and issues with subcellular localization, making them less than ideal for accurate and comprehensive ROS measurement. Additionally, commercially available ROS detection kits involve complex methodologies, and errors stemming from improper operation or suboptimal experimental conditions can lead to inaccurate or misleading conclusions [13]. Therefore, there is a pressing need for the development of highly sensitive, specific, and accessible ROS detection methods to facilitate more accurate and reproducible research outcomes.

Secondly, the inherent complexity and heterogeneity of ROS pose additional challenge in mechanistical clarification. ROS encompasses a diverse array of molecules, each with unique reactivity and biological roles [76,124]. On one hand, ROS detection often leads to a lack of specificity in experimental studies, obscuring mechanistic insights and complicating reproducibility [13]. On the other hand, ROS create a web of interactions that involve both inherent and ROS-modulated complexity in cancer cell signaling [527]. The relationship between ROS and signaling pathways is not straightforward, and the degree of ROS impact on various pathways is unclear [527,528]. In practice, the use of imprecise terms such as "low," "moderate," or "high" ROS levels further fails to capture the nuanced biological activities of distinct ROS species and convey objective conditions, thereby hindering accurate quantification and replication of experiments. Therefore, we call for the establishment of guidelines for the nomenclature and measurement of ROS, oxidative reactions, and oxidative damage.

Thirdly, precisely modulating ROS levels to selectively target tumor cells remains a formidable challenge in future translational research. Given the dual role of ROS in both promoting and inhibiting tumor growth [105,106], achieving an optimal ROS concentration is essential for effective RCD-based therapies. Insufficient ROS levels may inadvertently enhance tumor survival and proliferation [529,530], while excessive ROS could result in unintended cytotoxicity to surrounding normal tissues [105]. This delicate equilibrium underscores the necessity for personalized ROS modulation strategies, tailored not only to the tumor type but also to the specific cell death pathways involved.

Fourthly, rapid redox adaptation in cancer cells significantly limits the clinical efficacy of single-agent ROS-inducing therapies [7]. This adaptive resistance is mediated through multiple mechanisms, such as the activation of the NRF2 pathway [525], metabolic shifts towards NADPH production [526], and intercellular antioxidant transfer via exosomes [531]. These adaptations collectively enable tumor cells to evade ROS-mediated cytotoxicity, necessitating combinatorial targeting strategies [7,532]. For example, in chronic lymphocytic leukemia (CLL) cells, the combination of the ROS inducer arsenic trioxide and the SOD inhibitor 2-Methoxyestradiol exhibits potent antitumor activity, even in cases where CLL cells are resistant to 2-Methoxyestradiol monotherapy [533]. Similarly, the combined administration of ascorbic acid, which depletes intracellular GSH, and arsenic trioxide markedly enhances cytotoxic effects against drug-resistant multiple myeloma cells [534]. However, due to the inherent flexibility and reversibility of redox adaptation networks, along with substantial intertumoral and intratumoral redox heterogeneity, the effectiveness of combination therapies remains limited [535]. Therefore, the development of more precise, personalized therapeutic strategies capable of identifying and targeting specific vulnerabilities in individual tumors is a promising direction for future research, in contrast to uniform treatment approaches [535].

Finally, several chemotherapeutics such as paclitaxel [536], cisplatin [532], and doxorubicin [537], leverage ROS induction to initiate RCD in cancer cells, while these drugs were initially developed for other therapeutic purposes, thus leading to unavoidable toxic and side effects. Likewise, several compounds selectively targeting specific redox pathways to promote ROS-mediated RCD in cancer cells have been identified [366,524]. However, many of them were not initially developed to kill tumor [366,395]. Repurposing these agents for anticancer applications is still a long way to go because their safety and effectiveness in cancer patients are unknown [410,441,457]. Therefore, translating ROS-mediated RCD-inducing agents into clinical practice entails significant hurdles.

In conclusion, this review has examined the dualistic role of ROS in tumors, their regulatory influence on cancer cell signaling pathways, and the mechanisms by which ROS modulate RCD, highlighting the therapeutic potential of targeting ROS-mediated RCD in oncology. However, this field is fraught with complex challenges that demand rigorous, multidimensional research efforts and comprehensive evaluation to facilitate successful clinical translation.

## CRediT authorship contribution statement

**Danyao Chen:** Writing – original draft. **Ziyu Guo:** Writing – original draft. **Lei Yao:** Funding acquisition, Writing – original draft. **Yuming Sun:** Methodology. **Yating Dian:** Methodology. **Deze Zhao:** Methodology. **Yizhe Ke:** Methodology. **Furong Zeng:** Conceptualization, Funding acquisition. **Chunfang Zhang:** Methodology. **Guangtong Deng:** Conceptualization, Funding acquisition. **Linfeng Li:** Conceptualization, Funding acquisition.

## Availability of data and materials

Data and material will be deposited and publicly available.

## Ethics approval and consent to participate

N/A.

## Consent for publication

The corresponding author has received consent for publication.

## Funding

This work was supported by the 10.13039/501100001809National Natural Science Foundation of China (Grant Nos. 82301999 to LL, 82272849 to GD, 82403139 to LY), Huxiang Youth Talent Program (Grant Nos. 2023RC3072 to 10.13039/100004690GD, 2024RC3043 to FZ), Natural Science Fund for Outstanding Youths in Hunan Province (Grant Nos. 2023JJ20093 to GD), Postdoctoral Fellowship Program of CPSF (Grant Nos. GZC20242053 to LY), Changsha Municipal Natural Science Foundation (Grant Nos. kq243010 to LY), and 10.13039/501100011790Xiangya Hospital Youth Fund (Grant Nos. 2023Q17 to LY).

## Declaration of competing interest

The authors declare that they have no known competing financial interests or personal relationships that could have appeared to influence the work reported in this paper.

## Data Availability

No data was used for the research described in the article.

## ABBREVIATIONS

**ATM**: Ataxia telangiectasia mutated
**ATR**: Ataxia telangiectasia and Rad3 related
**ATO**: Arsenic Trioxide
**CAT**: Catalase
**DNA-PK**: DNA-dependent protein kinase
**ER**: Endoplasmic reticulum
**ERAD**: ER-associated degradation
**ERO1**: Endoplasmic reticulum oxidoreductase 1
**FDX1**: Ferredoxin 1
**GPX4**: Glutathione peroxidase 4
**GPX7/GPX8**: Glutathione peroxidase 7/8
**GSH**: Glutathione
**HIF-1α**: Hypoxia-inducible factor 1-alpha
**JNK**: c-Jun N-terminal kinase
**Keap1**: Kelch-like ECH-associated protein 1
**MPO**: Myeloperoxidase
**NE**: Neutrophil elastase
**NOXs**: NADPH oxidases
**NRF2**: Nuclear factor erythroid 2-related factor 2
**PDI**: Protein disulfide isomerase
**PRDX4**: Peroxiredoxin 4
**RCD**: Regulated cell death
**ROS**: Reactive oxygen species
**SLC7A11**: Solute carrier family 7 member 11
**SOD**: Superoxide dismutase
**Trx**: Thioredoxin
**TXNI****P**: Thioredoxin interacting protein
**TXNRD**: Thioredoxin reductase
**UPR**: Unfolded protein response
